# Genomic insights into the evolution and adaptation of secondary metabolite gene clusters in fungicolous species *Cladobotryum mycophilum* ATHUM6906

**DOI:** 10.1093/g3journal/jkae006

**Published:** 2024-01-12

**Authors:** Anastasia C Christinaki, Antonis I Myridakis, Vassili N Kouvelis

**Affiliations:** Section of Genetics and Biotechnology, Department of Biology, National and Kapodistrian University of Athens, Athens 15771, Greece; Section of Genetics and Biotechnology, Department of Biology, National and Kapodistrian University of Athens, Athens 15771, Greece; Section of Genetics and Biotechnology, Department of Biology, National and Kapodistrian University of Athens, Athens 15771, Greece

**Keywords:** *Cladobotryum mycophilum*, genome, fungicolous fungi, biosynthetic gene cluster (BGC), secondary metabolism, horizontal gene transfer (HGT)

## Abstract

Mycophilic or fungicolous fungi can be found wherever fungi exist since they are able to colonize other fungi, which occupy a diverse range of habitats. Some fungicolous species cause important diseases on Basidiomycetes, and thus, they are the main reason for the destruction of mushroom cultivations. Nonetheless, despite their ecological significance, their genomic data remain limited. *Cladobotryum mycophilum* is one of the most aggressive species of the genus, destroying the economically important *Agaricus bisporus* cultivations. The 40.7 Mb whole genome of the Greek isolate ATHUM6906 is assembled in 16 fragments, including the mitochondrial genome and 2 small circular mitochondrial plasmids, in this study. This genome includes a comprehensive set of 12,282 protein coding, 56 rRNA, and 273 tRNA genes. Transposable elements, CAZymes, and pathogenicity related genes were also examined. The genome of *C. mycophilum* contained a diverse arsenal of genes involved in secondary metabolism, forming 106 biosynthetic gene clusters, which renders this genome as one of the most BGC abundant among fungicolous species. Comparative analyses were performed for genomes of species of the family Hypocreaceae. Some BGCs identified in *C. mycophilum* genome exhibited similarities to clusters found in the family Hypocreaceae, suggesting vertical heritage. In contrast, certain BGCs showed a scattered distribution among Hypocreaceae species or were solely found in *Cladobotryum* genomes. This work provides evidence of extensive BGC losses, horizontal gene transfer events, and formation of novel BGCs during evolution, potentially driven by neutral or even positive selection pressures. These events may increase *Cladobotryum* fitness under various environmental conditions and potentially during host–fungus interaction.

## Introduction

Hypocrealean fungi present a plethora of biological interactions in the environment, exhibiting various symbiotic modes of life, positively or negatively affecting the host (Redman *et al*. 2001). Some of them, especially species belonging to the family Hypocreaceae, established a fungicolous (also known as mycophilic) mode of life, due to their stable association with other fungi (Gams *et al*. 2004). Their hosts are principally Basidiomycetes; however, in certain cases, it has been found to be ascomycetes (Gams *et al*. 2004). Some Hypocrealean species are the most important mycopathogens of cultivated mushrooms causing pathological conditions, i.e. cobweb (*Cladobotryum* spp.), dry bubble (*Lecanicillium fungicola*), green mold (*Trichoderma aggressivum*), and wet bubble disease (*Mycogone* spp.; Gea *et al*. 2021). The rate of the reported infections is increased in a wide range of edible mushrooms, i.e. *Agaricus bisporus*, *Pleurotus ostreatus*, *Pleurotus eryngii*, *Ganoderma sichuanensis*, *Flammulina velutipes*, and *Hypsizygus marmoreus* (Grogan and Gaze 2000; Back *et al*. 2012; Zuo *et al*. 2016; Gea *et al*. 2019).

*Cladobotryum* species are highly adaptable with a broad host range, affecting the sporophores of cultivated and wild Basidiomycetes, but also those of ascomycetes (Adie *et al*. 2006; Carrasco *et al*. 2017; Lakkireddy *et al*. 2020; Milic *et al*. 2022). *Cladobotryum mycophilum*, *Cladobotryum dendroides*, *Cladobotryum protrusum*, and *Cladobotryum varium* are some of the most common pathogenic agents in mushroom farms. *Cladobotryum mycophilum* is a highly important member, reported to cause cobweb disease primarily on Agaricales including the cultivated strains of white button (*A. bisporus*) and king oyster (*P. eryngii*) mushrooms (Gea *et al*. 2011, 2012; Kim *et al*. 2012; Muhammad *et al*. 2019; Milic *et al*. 2022). The sexual state of certain *Cladobotryum* species is associated with the genus *Hypomyces*, while for the rest, their anamorphs (conidial or asexual states) are not correlated with any teleomorphs (Rehner and Samuels 1995). Studies of *C. mycophilum*, the anamorph of *Hypomyces odoratus*, are focused on its diversity, distribution, and phylogenetic positioning within the genus, using gene regions of the internal transcribed spacer (ITS), the RNA polymerase II subunit (RPB1 and RPB2), and the translation elongation factor 1-alpha (TEF1) for barcoding, in addition to secondary metabolite profiling (Põldmaa 2011; Milic *et al*. 2022).

The genomic analyses of *Cladobotryum* species and in extent the genomes' contribution to the biotechnological importance of this genus are currently in need, since finding new environmentally friendly solutions for preventing mushroom cultivation damage is fundamental. Additionally, the potential of *C. mycophilum* as a biological control agent against fungal phytopathogens has been examined, and the initial outcomes appeared promising (Santos *et al*. 2019). All these factors necessitate the genetic elucidation of fungal–fungal interactions. To date, there is limited knowledge regarding the genetic mechanisms underlying *Cladobotryum*'s colonization in respective hosts, which is crucial for enhancing cultivation efficiency through environmentally friendly methodologies.

Filamentous fungi produce a wide range of secondary metabolic compounds, which play a crucial role in fungal development and shape their interactions of fungi with other organisms (Keller 2019; Zhang *et al*. 2020). In some cases, the production of these metabolites is associated with host colonization and their mode of life (Lysøe *et al*. 2011; Pedrini 2018; Cheng *et al*. 2020; Henríquez-Urrutia *et al*. 2022). The genus *Cladobotryum* is notable for its abundant and diverse secondary metabolism, including the production of red pigments (Põldmaa 2011), cladobotric acids G–I (Dao *et al*. 2022), cyclopeptides (Zhou *et al*. 2021), parnafungins and their open-ring forms (Bills *et al*. 2009), flavipucine and brunnescin (Wagner *et al*. 1995), and various other secondary metabolites (Milic *et al*. 2022). In contrast to primary metabolite synthesis, genes responsible for the production of secondary metabolites are often organized in contiguous biosynthetic gene clusters (BGCs) (Bradshaw *et al*. 2013; Keller 2019). BGC genes encode synthases, tailor enzymes, transcription factors, proteins with toxic properties, and other regulatory functions. BGCs are often coregulated, controlling the production of the secondary metabolites, and as a result, they play an important role in species ecological functions (Keller 2019; Gotting *et al*. 2022).

The elucidation of fungicolous interactions through the identification of secondary metabolic pathways and other pathogenicity-related genes can be achieved by comparative genomics. Until now, the genomes of *C. dendroides* and *C. protrusum* along with the causal agent of wet bubble disease in *A. bisporus*, i.e. *Mycogone perniciosa*, are available, revealing a diverse array of secondary metabolic BGCs (Li *et al*. 2019; Sossah *et al*. 2019; Xu *et al*. 2020). Identification of BGCs implicated to the secretion of secondary metabolic compounds, pathogen–host interaction (PHI) genes, transposable elements (TEs), carbohydrate-active enzymes (CAZymes), secretory proteins, membrane transport proteins, and cytochromes P450 will provide the necessary information in order to understand the mycophilic mode of life of *C. mycophilum*. Comparative genomics are essential to reveal the molecular mechanisms and processes involved in the fungicolous mode of life in Hypocreales, illuminating its evolutionary ancestry.

The present work entails the de novo sequencing of the whole genome of the Greek isolate *C. mycophilum* ATHUM6906, utilizing Nanopore technology, for the purposes of (1) presenting a high-quality reference genome for *C. mycophilum* ATHUM6906, (2) identifying all genes and genetic elements potentially related to mycophilic behavior, (3) exploring its secondary metabolic range and plasticity, and (4) conducting a comparative secondary metabolism analysis in genomic and in extend evolutionary level, when the existing genomes of species within the Hypocreaceae family are under scrutiny. Overall, the current effort establishes the groundwork for a deeper understanding of the fungicolous adaptation of *C. mycophilum* and in extent of genus *Cladobotryum*, with the future goal to employ this knowledge for biocontrol of mushroom's cobweb disease.

## Materials and methods

### Fungal material and culture conditions

*Cladobotryum mycophilum* ATHUM6906 was collected from *Hypholoma* sp., a species belonging to the order Agaricales, in *Castanea* sp. forest, Mt. Pilio, Magnisia, Greece, 2009 (Milic *et al*. 2022). The fungus was deposited at the Mycetotheca ATHUM in the Dried Specimen Collection and Culture Collection of Fungi of the National and Kapodistrian University of Athens (NKUA, Athens, Greece). The isolate was cultured in Petri dishes (diameter: 90 mm) on potato dextrose broth with 1% yeast extract, incubated in a shaker (200 rpm) at 25°C in a natural day/night photoperiod. Preliminary in-house experiments showed that it is capable of infecting cultivations of the edible mushroom *A. bisporus*.

### DNA extraction and sequencing

Mycelium was collected by vacuum filtration, and the total DNA isolation was performed using 100 mg of fungal material, using the HigherPurity Plant DNA Purification Kit (Canvax Reagents S.L., Valladolid, Spain) according to manufacturer's instructions. The extracted DNA was checked for quality and quantity using a NanoDrop (Thermo Fisher Scientific, Waltham, MA) and the Qubit broad range DNA assay kit (Thermo Fisher Scientific Waltham, MA), respectively.

One microgram of the total genomic DNA was used for Nanopore library preparation using the 1D Ligation Sequencing Kit (SQK-LSK110, Oxford Nanopore Technologies, Oxford, UK). Sequencing was performed using the R9.4.1 flow cell on a MinION device (Oxford Nanopore Technologies, Oxford, UK). Base calling was performed offline with ONT's Guppy software pipeline version 3.4.5, enabling the --pt_scaling flag and setting the --trim_strategy flag to DNA.

### Long read filtering, correction, and assembly

Adapter trimming of the raw sequences was performed by Porechop version 0.2.4 (www.github.com/rrwick/Porechop), setting the --adapter_threshold to 96 and enabling the --no_split flag. Setting the genome size to 40 Mb, the trimmed reads were further trimmed and corrected using Canu version 2.2 (Koren *et al*. 2017), enabling the -trim and -correct flags, respectively. Genome assembly was created using the Flye version 2.9.1 (Kolmogorov *et al*. 2019) using the --nano-corr flag, setting the genome size to 40 Mb and enabling the --trestle flag. Bandage v.0.9.0 (Wick *et al*. 2015) was used to visualize assembly graphs and search for telomere sequences by using the built-in blast function to search the telomere sequence (TTAGGGT)n_5–15_. In order to evaluate the completeness of the final genome assembly, Benchmarking Universal Single-Copy Orthologs (BUSCO) analyses were performed with BUSCO version 5.4.7, using hypocreales_odb10 lineage gene set (Manni *et al*. 2021).

### Gene prediction and functional annotation

Assembly annotations were performed using GenSAS v.6.0 (Humann *et al*. 2019), unless otherwise stated. Interspersed repeats, low complexity DNA sequences, and TEs were detected and masked by RepeatModeler v.2.0.1 and RepeatMasker v.4.1.1 (http://www.repeatmasker.org/) setting the DNA source to fungi. RNAmmer version 1.2 (Lagesen *et al*. 2007) and tRNAscan-SE version 2.0.7 (Lowe and Chan 2016) were used to detect the ribosomal RNA and tRNA genes, respectively. In order to primarily identify genomic regions with putative protein genes, transcript alignments were performed with BLAST nucleotide (blastn) tool version 2.12.0 using transcript database NCBI refseq fungi (Camacho *et al*. 2009) and BLAT tool version v2.5 using Transcripts FASTA file: NCBI refseq fungi (Kent 2002) and protein alignments were performed using DIAMOND proteins version 2.0.11 against Protein Data Set: NCBI refseq fungi (Protein; Buchfink *et al*. 2021). De novo gene prediction was performed using the following tools with default parameters: (1) AUGUSTUS tool version S3.4.0 with the reference gene data set of *Fusarium graminearum* (Stanke and Morgenstern 2005), (2) GeneMarkES version 4.48 (Ter-Hovhannisyan *et al*. 2008), and (3) GlimmerM tool version 2.5.1 selecting *Aspergillus* reference organism (Delcher *et al*. 2007). The tool EvidenceModeler was used to create a consensus gene set using the output files of the previously distributed tools (Haas *et al*. 2008). The ab initio official gene set (OGS) was evaluated with BUSCO analysis. Functional analysis of the OGS was performed using (1) BLAST protein vs protein (blastp) against Protein Data Set: NCBI refseq fungi (Protein; Camacho *et al*. 2009), (2) DIAMOND Functional version 2.0.11 against Protein Data Set: NCBI refseq fungi (Protein; Buchfink *et al*. 2021), and (3) InterProScan version 5.53-87.0 (Jones *et al*. 2014). The presence and location of signal peptide cleavage sites in amino acid sequence were identified using SignalP version 5.0b setting the -org flag to eukaryote (Petersen *et al*. 2011). The annotation of the OGS was performed using local BLASTp (*e*-value 1 × 10^−50^) against nonredundant (NR) protein sequence (Sayers *et al*. 2022), Swiss-Prot (Bateman *et al*. 2017), KEGG (Kanehisa *et al*. 2016), Gene Ontology (GO; Harris *et al*. 2004), clusters of orthologous groups for eukaryotic complete genomes (COG; Harris *et al*. 2004), PHI (Urban *et al*. 2022), CAZymes (Drula *et al*. 2022), MEROPS (Rawlings *et al*. 2014), PredGPI prediction server (Pierleoni *et al*. 2008), and Transporter Classification (TCdb; Saier *et al*. 2021) databases. TMHMM v2.0 was used to identify transmembrane proteins based on a hidden Markov model for transmembrane helices (Krogh *et al*. 2001).

### Mitochondrial DNA and mating-type idiomorph characterization

The mitochondrial contig was annotated as follows: the protein coding, ribosomal (rRNA), and tRNA genes were identified using BLASTx, BLASTn (Johnson *et al*. 2008), and tRNAscan-SE version 2.0.7 (Lowe and Chan 2016), respectively. The genetic code employed was “The Mold, Protozoan, and Coelenterate Mitochondrial Code and the Mycoplasma/Spiroplasma Code” (NCBI transl_table=4). The mitochondrial genome and plasmid were visualized using OrganellarGenomeDRAW (OGDRAW) version 1.3.1 (Greiner *et al*. 2019). The mating-type genes for *C. mycophilum* were determined by tBLASTx against the respective ones of the related species *C. dendroides* and *C. protrusum* (Sossah *et al*. 2019; Xu *et al*. 2020), and this locus was visualized by Lasergene's MegAlign v.11 program (Burland 2000).

### Comparative genomics

To study the evolution and genetic diversity of *C. mycophilum*'s genome, OrthoVenn3 tool (Sun *et al*. 2023) was used to identify and annotate orthologous clusters and infer phylogenetic relationships among selected species representing Hypocreaceae family, i.e. *Trichoderma harzianum*, *Trichoderma virens*, *C. dendroides*, *C. protrusum*, *Escovopsis* sp., and *Hypomyces perniciosus*, with the entomopathogenic species *Beauveria bassiana* used as outgroup (NCBI assembly accessions: GCF_003025095.1; GCF_000170995.1; GCA_011799845.1; GCA_004303015.1; GCA_003055185.1; GCA_008477525.1; and GCA_000280675.1, respectively). The OrthoFinder algorithm (Emms and Kelly 2019) was selected enabling annotation, protein similarity analysis, and cluster relationship network, using default parameters. Maximum likelihood phylogenetic analysis of the selected species was also conducted in OrthoVenn3 interference using the program FastTree2 and the evolution model JTT + CAT (Price *et al*. 2010), and the reliability of each node was determined by the Shimodaira-Hasegawa (SH) test. CAFE5 Software was used to calculate the contraction and expansion in gene family size (Mendes *et al*. 2021). The divergence times were set 133 million years ago (MYA) for *B. bassiana* and *T. harzianum* and 76 MYA among *Escovopsis* sp. and *T. harzianum*, as found in TimeTree 5 web portal (Kumar *et al*. 2022). Synteny analyses among ATHUM6906 and the genomes of *C. dendroides* and *C. protrusum* were performed comparing using i-ADHoRe3 (Proost *et al*. 2012), with default parameters, and the visualization was performed locally using Circos version 0.69-9 (Krzywinski *et al*. 2009).

### BGCs and secondary metabolism comparative analysis

Forty genomes from species belonging to Hypocreaceae family were selected for comparative analysis of secondary metabolism (Table 1). In all species, BGCs were identified using antiSMASH 7.0 (Blin *et al*. 2023) enabling the cluster border prediction based on transcription factor binding sites (CASSIS) selection. All genes that were identified as part of BGCs were analyzed in OrthoFinder v2.5.5 (Emms and Kelly 2019), in order to examine the shared and unique secondary metabolism–related protein-coding genes (PCGs) and BGCs in Hypocreaceae family. The rooted species tree was created setting the “-M msa” parameter, which enables MAFFT to create multiple sequence alignments and FastTree to construct a maximum likelihood secondary metabolism–based tree. The similarity network analysis and the exploration of BGC diversity within Hypocreaceae family were conducted using the BiG-SCAPE program (Navarro-Muñoz *et al*. 2020). BiG-SCAPE is a genome mining designed for the rapid and interactive examination of BGCs across multiple genomes, facilitates the development of similarity networks, and enables the classification of BGCs into families. To identify similarities with known metabolites, all reference BGCs from the MIBiG database were included in the analysis (Terlouw *et al*. 2023). Horizontal gene transfer (HGT) analysis and Alien Index (AI) scores of candidate HGTs.

**Table 1.** Selected species and strains belonging to Hypocreaceae family, included in the secondary metabolism comparative analysis of this study.

Number	Species	Strain	GenBank assembly accession
1	*Cladobotryum dendroides*	CCMJ2808	GCA_011799845.1
2	*Cladobotryum mycophilum^a^*	ATHUM6906	JAVFKD000000000
3	*Cladobotryum protrusum*	CCMJ2080	GCA_004303015.1
4	*Escovopsis* sp.	TC	GCA_003055185.1
5	*Escovopsis* sp.	Ae724	GCA_003055165.1
6	*Escovopsis weberi*	—	GCA_001278495.1
7	*Hypocreaceae* sp.	CTeuk-1143	GCA_029290895.1
8	*Hypomyces perniciosus*	HP10	GCA_008477525.1
9	*Mycogone perniciosa*	MgR1	GCA_025331125.1
10	*Sphaerostilbella broomeana*	TFC201724	GCA_930272545.1
11	*Trichoderma afroharzianum*	IIPRTh-33	GCA_020736905.1
12	*Trichoderma arundinaceum*	TP19.13	GCA_025919625.1
13	*Trichoderma asperelloides*	T203	GCA_021066465.1
14	*Trichoderma asperellum*	CBS 433.97	GCA_003025105.1
15	*Trichoderma atrobrunneum*	ITEM 908	GCA_003439915.1
16	*Trichoderma atroviride*	IMI 206040	GCA_000171015.2
17	*Trichoderma breve*	T069	GCA_028502605.1
18	*Trichoderma brevicompactum*	IBT 40841	GCA_003012085.1
19	*Trichoderma brevicrassum*	TC967	GCA_017311225.1
20	*Trichoderma citrinoviride*	TUCIM 6016	GCA_003025115.1
21	*Trichoderma cornu-damae*	KA19-0412C	GCA_020631695.1
22	*Trichoderma erinaceum*	CRRI-T2N1	GCA_013365115.1
23	*Trichoderma gamsii*	T6085	GCA_001481775.2
24	*Trichoderma gracile*	HK011-1	GCA_020002365.1
25	*Trichoderma hamatum*	GD12	GCA_000331835.2
26	*Trichoderma harzianum*	CBS 226.95	GCA_003025095.1
27	*Trichoderma koningii*	JCM 1883	GCA_001950475.1
28	*Trichoderma koningiopsis*	RA3a	GCA_022985005.1
29	*Trichoderma lentiforme*	CFAM-422	GCA_011066345.1
30	*Trichoderma lixii*	MUT 3171	GCA_014468695.1
31	*Trichoderma longibrachiatum*	FL-4	GCA_026259275.1
32	*Trichoderma oligosporum*	CGMCC 3.17527	GCA_015266385.1
33	*Trichoderma parareesei*	CBS 125925	GCA_001050175.1
34	*Trichoderma pleuroti*	TPhu1	GCA_001721665.1
35	*Trichoderma pseudokoningii*	—	GCA_943193705.1
36	*Trichoderma reesei*	QM6a	GCA_000167675.1
37	*Trichoderma semiorbis*	FJ059	GCA_020045945.2
38	*Trichoderma simmonsii*	GH-Sj1	GCA_019565615.1
39	*Trichoderma virens*	Gv29-8	GCA_000170995.1
40	*Trichoderma viride*	Tv-1511	GCA_007896495.1

Their GenBank assembly accession numbers are also provided.

^*a*^Current work.

In order to determine if the core genes of the NI-siderophore, type 3 polyketide synthase (T3PKS), and non-alpha poly-amino acid (NAPAA) BGCs, which were found only in *Cladobotryum* genomes, are a result of HGT, the analyses described below were performed.

For each BGC core gene, a Blastp search was performed against the NR database, with default parameters. The first 250 hits with the highest score (*e*-value and identity) were included in the creation of an amino acid alignment. All alignments were acquired using the MAFFT online tool (Katoh *et al*. 2019) using default parameters. As a result, 3 matrices were created, henceforward called “NI-siderophore-matrix,” “T3PKS-matrix,” and “NAPAA-matrix” for NI-siderophore, T3PKS, and NAPAA core biosynthetic genes, respectively. For each matrix, amino acid model selection was performed in IQ-TREE (Kalyaanamoorthy *et al*. 2017). Maximum likelihood phylogenetic trees were constructed in IQ-TREE with the ultrafast bootstrap with the selected amino acid model (Nguyen *et al*. 2015).

In order to define the origin of the terpene synthase gene (cl_my.00g000130), a Blastp search was performed against the NR database. Primarily, the first aligned amino acid sequences with the highest score (*e*-value and identity) belonged to Basidiomycetes. In order to ensure that the results are true and independent, a second search excluding sequences derived from Basidiomycetes was performed. As a result, 250 sequences of Basidiomycetes and 250 sequences of other fungi were selected to produce the “terpene-matrix.” Additionally, Hypocreaceae terpene synthases, which were identified in the current work, were included. The “terpene-matrix” and phylogenetic tree was constructed as described previously.

To assess potential HGT events, the AI score was calculated for all candidate HGT genes included in the BGCs mentioned above. This quantitative method was employed to measure how well the *C. mycophilum* protein sequences align with those from non-Hypocrealean vs Hypocrealean. The AI score for each gene was computed by utilizing the *e*-value derived from the best sequence alignment of *C. mycophilum* proteins against all Hypocrealean and non-Hypocrealean sequences present in the NCBI NR database. The formula proposed by Matriano *et al*. (2021) was applied to calculate the AI score, allowing for a comprehensive evaluation of potential gene transfer events.

## Results and discussion

### Genome features and gene prediction

Genome sequencing of *C. mycophilum* ATHUM6906 produced a total of 1,581 Mb clean data which were further assembled into a 40.7 Mb draft genome with 37× mean coverage and 47.8 GC % (Table 2). This consisted of 16 contigs with N50 of approximately 5.07 Mb. The completeness of the genome was evaluated by mapping the BUSCOs against fungi_odb10 and hypocreales_odb10 data sets. This analysis indicated a high-quality genome, identifying 97.4 and 96.7% complete fungal and hypocrealean BUSCOs, respectively.

**Table 2.** Whole genome sequencing statistics and genomic features of *C. mycophilum* ATHUM6906.

ATHUM6906 genome statistics and features	
Total length (bp)	40,679,719
Number of fragments	16
N50 (bp)	5,067,140
Largest fragment (bp)	7,644,443
Mean genome coverage	37
Number of predicted tRNA genes	273
Number of predicted rRNA genes	56
Number of predicted PCGs	12,282
Transposable elements (%)	3.72
Mitochondrial genome size (bp)	76,524
Mitochondrial plasmid size (bp)	11,721

**Fig. 1.**
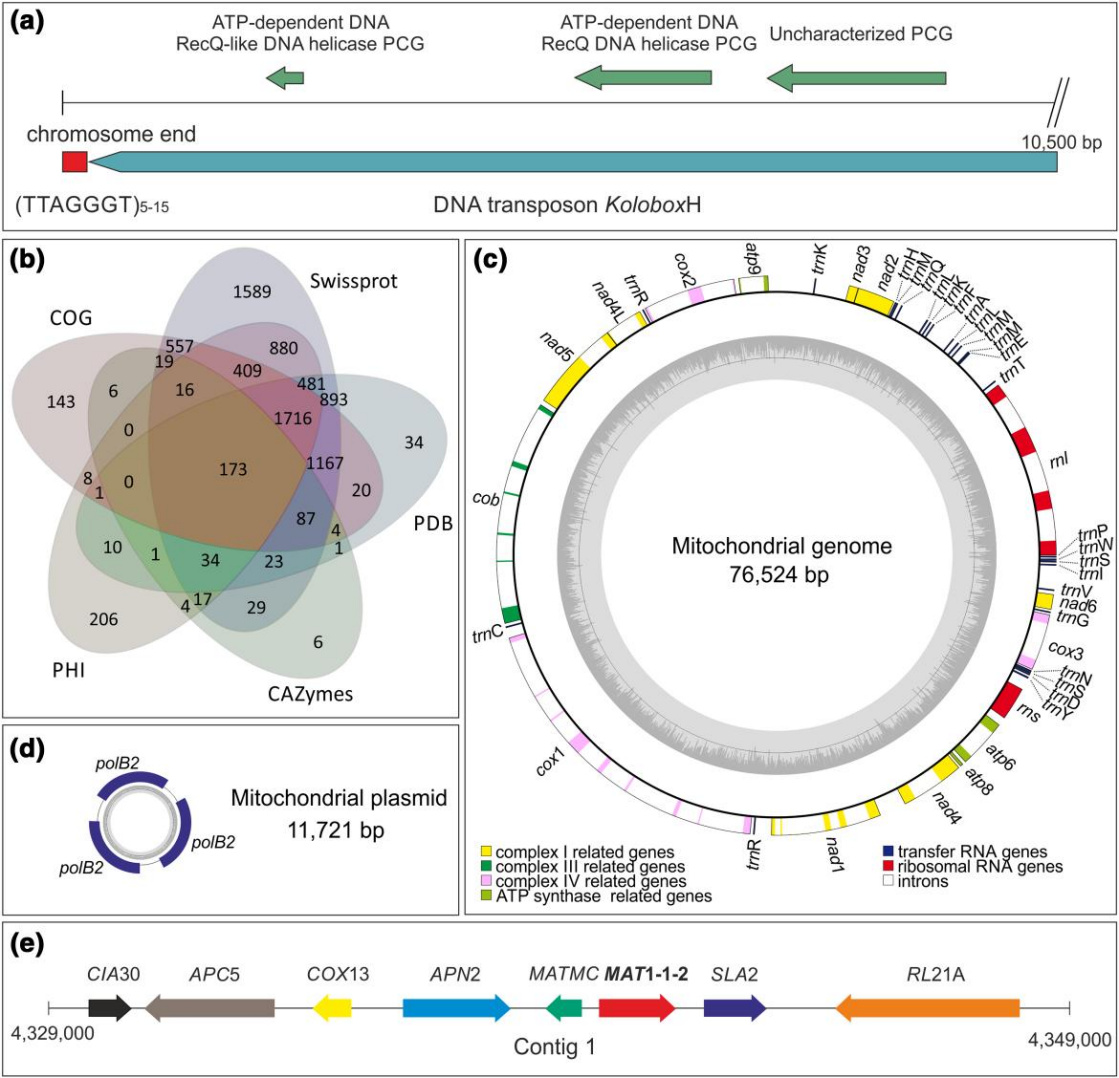
a) Structure of telomeres in the genome of *C. mycophilum* ATHUM6906, including the (TTAGGGT)_5–15_ telomeric repeat and the *Kolobox*H DNA transposon. b) Comparison of predicted PCG annotation results among Swiss-Prot, PDB, CAZy, PHI, and COG databases. c) The complete mitochondrial genomes of ATHUM6906. The genes related to complex I, III, IV, and ATP synthase, tRNA genes and rRNA genes are indicated in the mitochodrial genome map. d) The mitochondrial plasmid where 3 copies of *pol*B2 gene are located. e) Schematic representation of the structure of mating-type loci (*MAT 1-1-1*) in contig 1 of *C. mycophilum* ATHUM6906 genome. The arrows indicate the gene transcription orientation.

In filamentous fungi, the telomeric repeats were identified at the end of the linear chromosomes (Cohn *et al*. 2005). Among the 13 linear contigs of this genome, 8 contigs contained telomere structures at least at the one end and 9 telomeric sequences were identified in total. Three out of 16 contigs had circular topology (contigs 24, 28, and 29). In detail, the telomeres consisted of the (TTAGGGT)_n_ telomeric repeat followed by *Kolobok*H DNA transposon sequences, which presented sizes of ca. 10,470 bp (Fig. 1a). *Kolobok*H included 2 RecQ-like DNA helicase PCGs and an uncharacterized PCG, which is a similar structure with the one primarily found in a fungus of Glomeromycota, i.e. *Rhizophagus irregularis* (Kojima and Bao 2022). G-quadruplex (G4) analysis showed that the telomeric repeats of *C. mycophilum* could form G4 structures, which are implicated in telomere maintenance (Williamson *et al*. 1989; Zhao *et al*. 2010). Since 9 telomere structures were predicted, the genome of *C. mycophilum* consisted of at least 5 potential chromosomes. In *C. dendroides* genome, the 8 contigs are part of 6 potential chromosomes. Other Hypocrealean species, like *Trichoderma* spp. (3–11 chromosomes; Kubicek *et al*. 2019), *Metarhizium* spp. (7 chromosomes; Saud *et al*. 2021), *Epichloë* spp. (7 chromosomes; Treindl *et al*. 2021), and *Fusarium* spp. (4–15 chromosomes), possess from 3 to 15 haploid chromosomes.

Two hundred and seventy-one tRNA genes were located in almost all contigs with the exception of the 5 smallest fragments. On the contrary, the 56 identified rRNA genes are located in scaffolds 12 and 17 (Table 2).

A total of 12,282 PCGs were predicted by the combination of homology- and de novo–based gene prediction methods (Fig. 1b; Supplementary Table 1). Among them, 11,830 (96.31%) genes were homologous in the NCBI NR database, followed by InterPro database (11,089 genes—90.27%), Swiss-Prot database (8,090 genes—65.87%), KEGG database (4,074 genes—33.2%), GO database (8,033 genes—65.40%), COG database (4,326 genes—35.22%), PHI database (3,956 genes—32.21%), and CAZyme database (420 genes—3.42%). In *C. mycophilum* genome, 1,111 secretory proteins were predicted. TMHMM v2.0 predicted at least 1 transmembrane helix in 2,339 PCGs. Interestingly, according to TCdb, 1,004 genes were aligned with genes encoding transporters (Supplementary Table 1).

TEs were found on 3.72% of the *C. mycophilum* genome. Class I TEs (retroelements) are more abundant covering 2.14%, and most of them are classified as non-LTR (LINEs) at 2.13%, while LTRs make up 0.01% of the total genome (Supplementary File 1). Class II TEs are less common (0.95%) followed by unclassified repeats (0.63%). Similar coverage in TEs can be found in other species of the genus, i.e. *C. protrusum* (2.59%) and *C. dendroides* (4.37%), while in smaller genomes, TEs tend to be more abundant, like the fungicolous species *H. perniciosus* (23.25%) or *Escovopsis* sp. (5.89%; Li *et al*. 2019; Sossah *et al*. 2019). Eighteen percent (456 out of 2,469) of the TEs of the *C. mycophilum* genome were located within coding regions. Seventy percent of those TEs are located within a single exon while the rest span over multiple exons and introns.

### Circular molecules and identification of mating-type idiomorphs

The mitochondrial genome of *C. mycophilum* (contig 24; NCBI Acc. No: OP928225) was a circular molecule of 76,524 bp containing the 14 PCGs responsible for the oxidative phosphorylation and ATP production (*cox*1-3, *nad*1-6, *nad*4L, *cob*, *atp*6, and *atp*8-9), 2 rRNA genes (*rns* and *rnl*), and 26 tRNA genes (*trn*s; Table 2 and Fig. 1c). More than 54.68% of the mtDNA corresponded to intronic regions. In total, 28 introns of group I were identified, in most cases hosting GIY-YIG and LAGLIDADG homing endonuclease genes. Only *atp*8, *nad*2, *nad*3, *nad*6, and *rns* genes did not possess introns. High intraspecies variation was identified among *C. mycophilum* ATHUM6906 (76,524 bp) and the respective genome published by Chen *et al.* (2020) (78,529 bp—GenBank accession no. NC_054243.1). Although gene order is identical for these mitochondrial genomes, the one of ATHUM6906 was approximately 2 kb smaller (Chen *et al*. 2020). Intron indels and transpositions along with variation in their intergenic regions were identified. In detail, mt genes of strain ATHUM6906 had gained (i.e. in *nad*5, *cob*, *cox*1, *nad*1, *nad*4, and *rnl* genes) or lost intronic region (i.e. in *nad*2, *nad*3, *cox*2, *atp*6, and *cox*3) genes. Another circular molecule of 11,721 bp (contig 28) was identified (Table 2 and Fig. 1d). Based on its gene content, contig 28 seems to be a mitochondrial plasmid, according to its similarity with mt plasmids found in *Cryphonectria parasitica* and *Neurospora intermedia* (Hausner 2012). It contained the mitochondrial DNA polymerase gene in 3 copies, with high sequence identity to the respective gene of *C. parasitica*'s pCRY1 plasmid (Monteiro-Vitorello *et al*. 2000). This is a plasmid that reduces pathogenicity in *C. parasitica*, when present (Monteiro-Vitorello *et al*. 2000). Therefore, the *C. mycophilum* plasmid may play a similar role. However, more experiments for this plasmid have to be performed before concluding about its role. A third small circular molecule (contig 29) did not align with any known genes or genetic elements.

MAT1-1 mating-type idiomorph was identified in the genome of *C. mycophilum* ATHUM6906 in 1 copy located on contig 1, in agreement with the fungicolous species *C. dendroides* and in contrast to *C. protrusum* and *Hypomyces perniciosa*, which possess the MAT1-2 idiomorph (Fig. 1e). The existence of MAT1-1 mating type in strain ATHUM6906 does not exclude the possibility that both mating types may be present at *C. mycophilum* population level. Complex I intermediate–associated protein 30 kDa (*CIA*30), anaphase-promoting complex subunit 5 (*APC*5), cytochrome C oxidase subunit 13 (*COX*13), AP DNA endonuclease 2 (*APC*5), DNA-binding mating-type M-specific polypeptide Mc (MATMC), endocytosis protein end4 (*SLA*2), and S ribosomal protein L21-A (*RL*21A) genes were located upstream and downstream of MAT1-1-2 idiomorph, almost the same gene content compared to *C. dendroides*, *C. protrusum*, and *H. perniciosa* (Li *et al*. 2019; Sossah *et al*. 2019; Xu *et al*. 2020).

### Comparative genomics

**Fig. 2.**
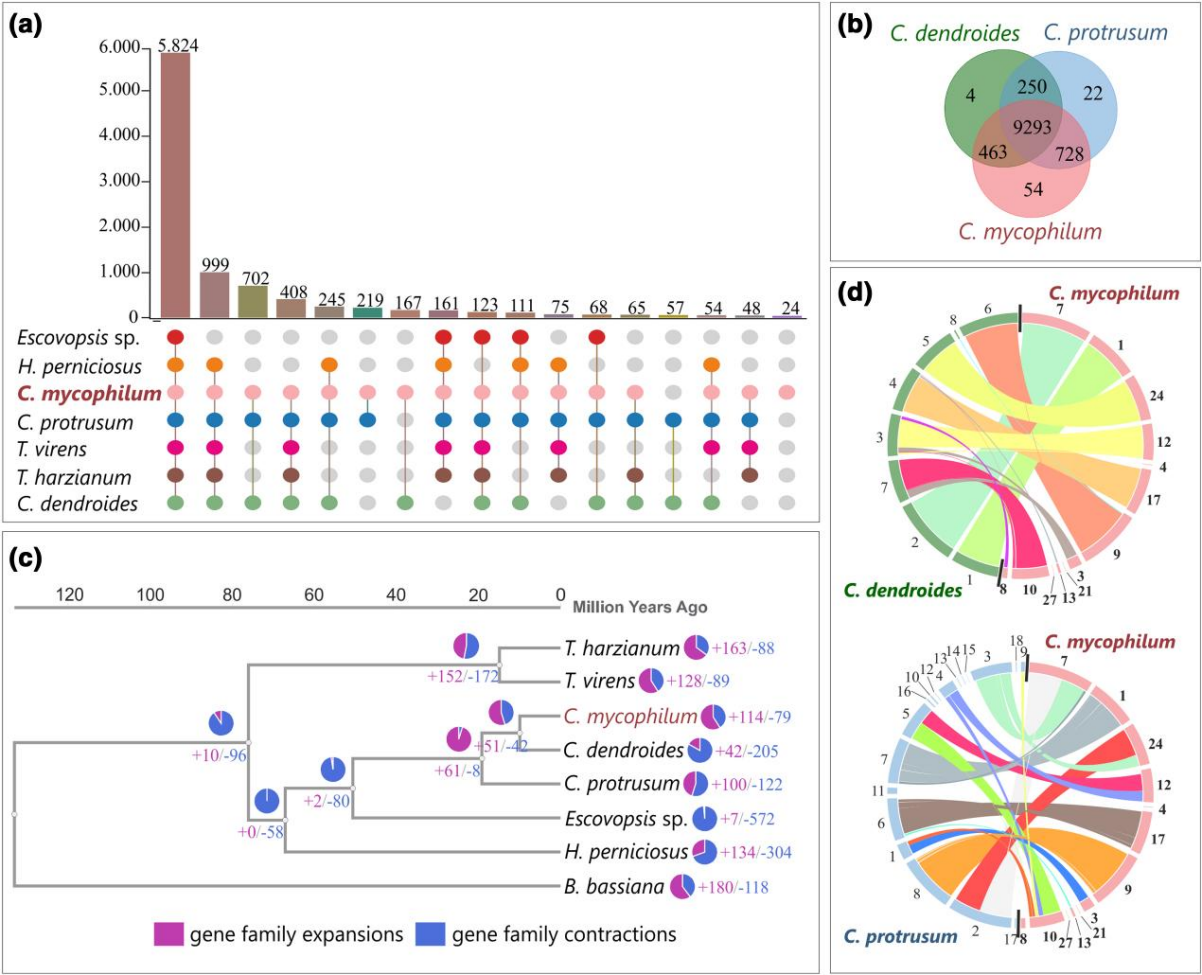
Genome comparison of *C. mycophilum* ATHUM6906. a) The chart presenting the number of orthologous clusters, which are shared among species. The numbers of orthogroups, which are shared within a group of species, as shown by their connected dots, are represented by the respective bar. b) The number of shared orthogroups among *Cladobotryum* species. c) The phylogenetic timetree of Hypocreaceae representatives based on their whole genome data. The tree was produced by the maximum likelihood method using the JTT + CAT evolutionary model. The numbers of contracted and expanded gene families are indicated in each node. d) Synteny comparison of *C. mycophilum* genome with *C. dendroides* (top) and *C. protrusum* (bottom). The Circo plots represent syntenic blocks. The outer numbers indicate chromosome numbers.

Comparative whole genome analysis among ATHUM6906 and other representatives of Hypocreaceae family, whose genomes are available and fully characterized, was performed to study the evolutionary changes among organisms and to identify the conserved and unique genes of the species examined (as described in M&M). Clustering all predicted PCGs among the representatives of Hypocreaceae family, 12,033 orthogroups were formed (Supplementary File 2). Interestingly, even though species are closely related, only half of the predicted orthogroups (i.e. 5,824) were shared in all examined species. This is due to *Escovospis* sp. Ae724, whose genome size is significantly smaller than the rest Hypocreaceae (approximately 30 Mb with only 7,231 PCGs). When genomic sequences of Ae724 were excluded, 999 more orthologous clusters were identified in the rest species examined, resulting in a total core set of 6,823 protein clusters (Fig. 2a). The PCGs of *C. mycophilum* were grouped in 9,883 clusters and 646 singletons. It shared more orthogroups with *C. protrusum* (728 clusters) than *C. dendoides* (463 clusters; Fig. 2b). Nevertheless, based on the current whole genome phylogenetic analysis, *C. mycophilum* was closer to *C. dendroides*, as they diverged 9.9 MYA, while *C. protrusum* was more phylogenetically distant, as it branched off 19.13 MYA (Fig. 2c). This positioning is also supported by other single locus-based phylogenetic analyses, in which the ITS was mainly used (Põldmaa 2011; Milic *et al*. 2022). One hundred and fourteen gene family expansions and 79 gene family contractions were found in *C. mycophilum*, related to genes with transferase activity, oxidoreductase activity, hydrolase activity, and other enzymatic or binding molecular functions (Fig. 2c; Supplementary Fig. 1). *Mycogone perniciosa* and *Cladobotryum* spp. (and their *Hypomyces* teleomorphs), both mushroom-associated fungicolous species sharing a variety of hosts, were diverged 67 MYA (*C. mycophilum*—*H. perniciosus*), forming a clade that also includes the fungicolous genus *Escovopsis* (Fig. 2c). According to the current comparative study, massive gene family contraction had happened during the formation of genus *Escovopsis* (i.e. 572 families), and only 7 gene families were expanded (Fig. 2a and c). Overall, the Hypocreaceae family, which includes mycophilic species, appears to diverge from the rest of Hypocreales approximately 130 MYA, which is in accordance with a respective study focused on *Trichoderma* spp. that estimated that this event had happened among 100–140 MYA (Kubicek *et al*. 2019).

*Cladobotryum* spp. appeared to share a core set of genes, if compared to other fungicolous species. The formation of *Cladobotryum* clade happened approximately 19.2 MYA, and it was accompanied by the expansion of 61 gene families. These unique families and protein effectors may be related to their adaptation in Agaricomycetes (Fig. 2c). Gene order was conserved among *C. mycophilum and C. dendroides* genomes, and only small syntenic blocks were transposed (Fig. 2d). According to chromosome 7 of *C. dendroides*, contigs 3 and 10, which both have telomeric sequences only in one end, may form a single chromosome. On the contrary, more transposition events were identified, when comparing *C. mycophilum* and *C. protrusum* gene order. For example, contig 7 of *C. mycophilum* consists of partial synteny blocks of contigs 2 and 3 of *C. protrusum*. Similar events were observed in contigs 10, 12, and 24, with contig 10 to include gene blocks from 4 contigs of *C. protrusum*.

### CAZymes and pathogenicity-related genes

In the first stages of infection, the pathogenic fungi use proteins of the CAZyme families to decompose the complex polysaccharides in the host cell wall (Bashyal *et al*. 2017). In *C. mycophilum*, 420 CAZymes were identified (Supplementary Table 1), with 214 genes encoding glycoside hydrolases (GHs), 97 genes encoding glycosyl transferases (GTs), 29 genes encoding carbohydrate-binding modules (CBMs), and 54 genes encoding auxiliary activities (AAs). From the 6 categories of CAZymes, the carbohydrate esterases (CEs; 12 genes) and polysaccharide lyases (PLs; 4 genes) were the most underrepresented. *Cladobotryum mycophilum* showed a significant abundance of CAZymes when compared to other species, which colonize basidiomycetous hosts like *A. bisporus*, i.e. 412 in *C. protrusum* (Sossah *et al*. 2019), 327 in *C. dendroides* (Xu *et al*. 2020), and 338 in *H. perniciosus* (Li *et al*. 2019). In the 4 fungicolous species mentioned above, GHs and GTs were the most commonly found enzymes, probably due to their role in the host cell wall degradation during the stages of infection.

The plethora of genes encoding cell wall–degrading enzymes is common in other fungicolous genera like *Trichoderma* in which genes encoding CAZymes were acquired through massive lateral transfer of genes from plant-associated fungi, a phenomenon which was not observed to the respective genes of *Escovopsis* sp. (Druzhinina *et al*. 2018; Kubicek *et al*. 2019). Druzhinina *et al.* (2018) did not include other fungicolous genera like *Cladobotryum* or *Mycogone*, due to the lack of genomic data at the time. According to Kubicek *et al.* in 2011, fungicolous behavior was an ancestral mode of life in genus *Trichoderma*, an ability lost in some species due to gene losses (Kubicek *et al*. 2011; Chaverri and Samuels 2013). All the above along with the plethora of CAZymes observed in *C. mycophilum*, *C. protrusum*, *C. dendroides*, and *H. perniciosus* suggest that the expansion in cell wall–degrading families may be related to the evolution of the fungicolous mode of life in Hypocreaceae.

Since chitin and β-(1,3) glucan are the core polysaccharides comprising fungal cell walls (Gow *et al*. 2017), chitinases and glucanases could be considered as core enzymes in host colonization process (Qin *et al*. 2020; Mart Nez-Cruz *et al*. 2021). In the genome of *C. mycophilum*, 26 chitinases were identified, all belonging to GH18 CAZyme category (Supplementary Table 2). An evolutionary study of chitinases showed that they are categorized in 3 groups, i.e. A, B, and C, with group B to be involved in fungicolous mode of life and entomopathogenicity of Sordariomycetes (Wang *et al*. 2021). Early-stage experiments in fungicolous fungi, like *T. harzianum*, have shown that some chitinases are upregulated during their interaction with a phytopathogenic fungus in vitro (Carsolio *et al*. 1994). This indicates the importance of GH18 chitinases in mycophilic behavior. Similarly, 37 PCGs were related to glucan degradation. These PCGs were found to be more variable since they belong to 13 different GH subfamilies and 1 AA family (Supplementary Table 3).

**Fig. 3.**
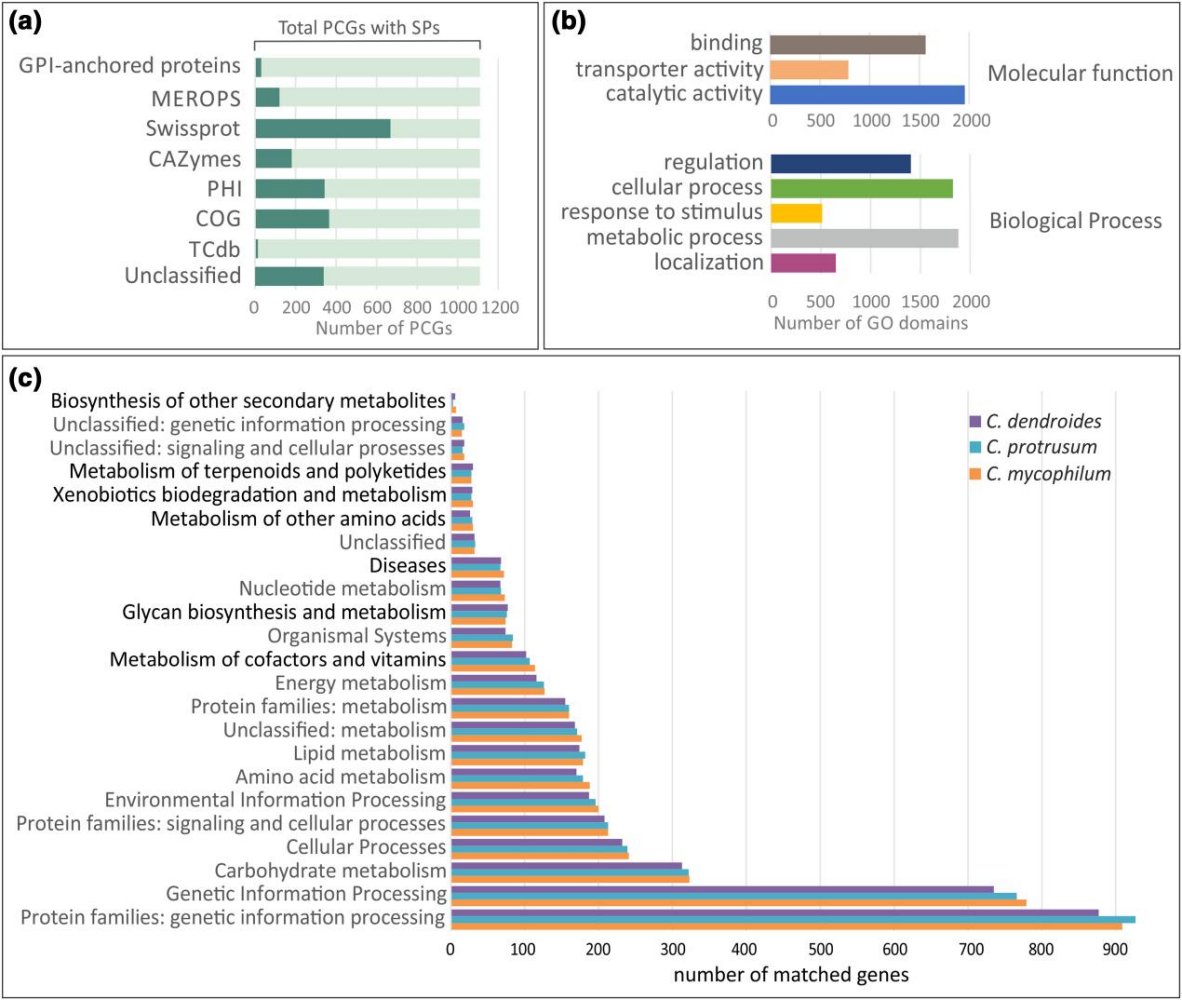
a) PCGs with secretory signal for secretory translocation found in each database compared to the total number of PCGs with SP signal for *C. mycophilum* ATHUM6906. b) Pathogenicity-related genes of *C. mycophilum* ATHUM6906, as found by PHI, and classified according to their molecular function (top) and their biological process (bottom) in GO database. PCGs with more than 1 biological domain received a GO number for each identified domain. c) Number of matched genes per cellular function of the primary and secondary metabolism among *Cladobotryum* species, as predicted in KEGG database, i.e. *Cladobotryum dendroides* (top bar), *C. protrusum* (middle bar), and *C. mycophilum* (bottom bar).

In fungi, secreted enzymes and other auxiliary proteins are highly important, especially in species that colonize the host surface (Bradley *et al*. 2022; Nazar Pour *et al*. 2022). In *C. mycophilum* genome, 1,111 secreted proteins were predicted (Supplementary Table 4), more than the respective ones in *C. protrusum* and *C. dendroides* genomes. Out of them, 339 PCGs had no match in any expertly curated database. From the rest of secretory proteins, 344 (31%) were predicted to encode pathogenicity–host-related genes (PHI), 181 (16.3%) CAZymes, 121 (10.9%) proteases (MEROPS database), and 31 GPI-anchored proteins (PredGPI prediction server), and only 15 genes were predicted to encode transporters (TCdb; Fig. 3a). In 15 chitinases and 18 glucan degradation enzymes, a Sec/SPI signal for secretory translocation was found, making them important candidates implicated in fungicolous mode of life.

Interestingly, according to PHI database, 32.21% PCGs of *C. mycophilum* ATHUM6906 could be related with pathogenicity (Supplementary Table 5). According to GO characterization, the identified biological domains of these putative pathogenicity-related genes were included in 3 functional categories (Fig. 3b). PCGs with binding and catalytic functions were the most abundant, as expected (Cairns *et al*. 2016). Furthermore, in *C. mycophilum*, more than 15% of pathogenicity-related genes encoded transporters highlighting their importance. Classified in respect to their biological process, most biological domains of pathogenicity-related PCGs were associated with metabolic processes of both primary and secondary metabolism (Fig. 3b). Preliminary analysis against KEGG database showed that more than 4,000 PCGs were associated with *C. mycophilum* primary and secondary metabolism, and 79 complete and 125 incomplete metabolic pathways were found. Some of them were implicated in biosynthesis of isoprenoids, the metabolism of cofactors and vitamins, in terpenoid, polyketides, and other secondary metabolite biosynthesis, and in xenobiotic biodegradation and metabolism (Fig. 3c). This indicated the necessity to further examine and characterize all genes related to the production of secondary metabolites.

### BGCs in *C. mycophilum*

**Fig. 4.**
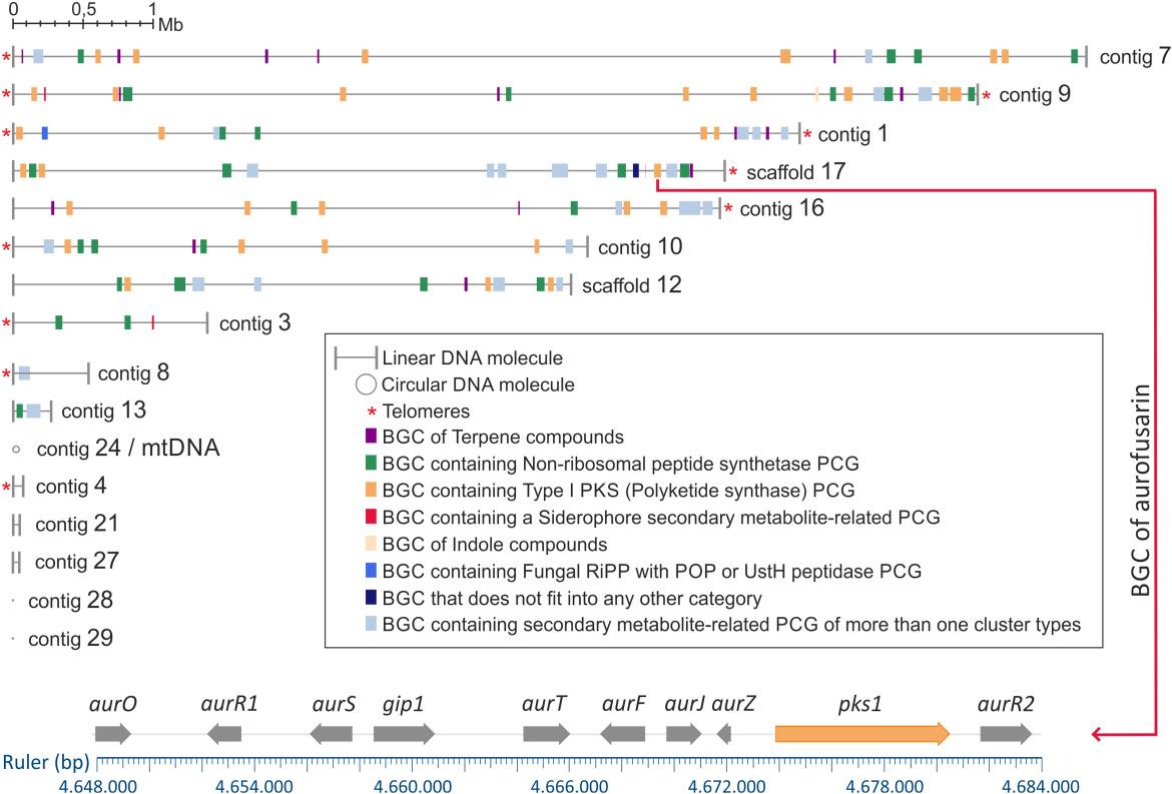
Schematic representation of secondary metabolite BGC location in the 16 predicted fragments of *C. mycophilum* genome. The size of BGCs of terpene, NRPS, T1PKS, siderophore, indole, fungal RiPP, other that does not fit into any other category, and clusters containing more than 1 type of synthase is proportional to the genome size. Asterisk (*) in the end of the DNA fragments indicates the presence of telomeric sequences. Genetic locus of aurofusarin T1PKS BGC in scaffold 17 is also presented in detail (bottom).

In fungi, multiple genes contributing to a secondary metabolic pathway are often clustered together in coregulated BGCs (Keller 2019). In *C. mycophilum* genome, 106 BGCs were identified, confirming its abundant secondary metabolism (Fig. 4). In total, 54 PCGs encoding type 1 polyketide synthases (T1PKSs), 48 nonribosomal polypeptide synthetases (NRPSs), 23 PCGs encoding terpene synthases, 3 fungal RiPP PCGs, 2 siderophore PCGs, 1 NAPAA like e-polylysine PCG, 1 indole PCG, and 1 T3PKS were found (Supplementary Table 6). In some cases, more than 1 synthase/synthetase genes from different categories were included in a single BGC (e.g. the NRPS-T1PKS BGC that has 72% similarity to BGC of chaetoglobosins from *Chaetomium globosum* CBS 148.51). In that case, clusters were overlapping each other, and in the common region, some regulatory or accessory genes exist and may play a role in both subclusters that form these BGCs. The shared auxiliary genes among these BGCs might possess a conserved nature, potentially serving as regulatory elements that have evolved from ancient fungal ancestors. This hypothesis could be an extension of the findings of a recent publication, which indicated that fungal genomes exhibit a delayed loss of ancestral gene families while rapidly duplicating certain gene groups, such as extracellular proteins and transcription factors (Merényi *et al*. 2023). Moreover, some BGCs had more than 70% similarity with well-known fungal biosynthetic clusters (i.e. chaetoglobosins, cichorine, wortmanamides A and B, clavaric acid, chrysogine, alternariol, dimethylcoprogen, pyranonigrin E, verticillin, naphthopyrone, aurofusarin, and depudecin).

**Fig. 5.**
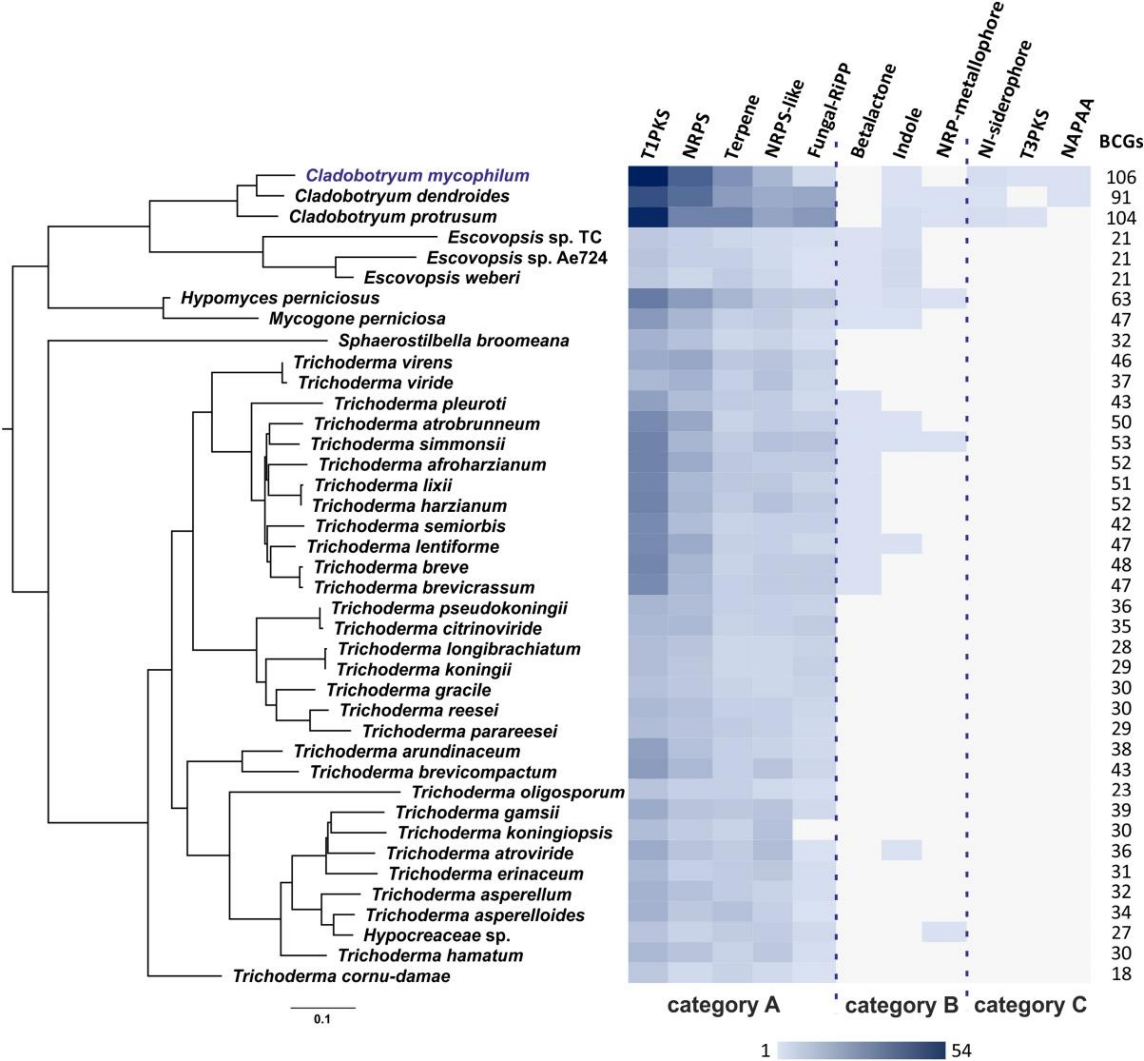
Heat map of BGC presence in 40 Hypocreaceae genomes in relation to their secondary metabolism phylogenetic tree. BGCs are grouped into 3 categories according to their distribution in the Hypocreaceae family. In detail, category A includes BGCs with wide distribution, category B BGCs with scattered distribution, and category C BGCs found only in *Cladobotryum* spp. The phylogenetic tree was produced by ML method in OrthoFinder v2.5.5 setting the “-M msa” parameter, which enables MAFFT to create multiple sequence alignments and FastTree to construct a maximum likelihood tree. All nodes are 100% supported. The number of the total BGCs of each species is indicated on the right.

Notably, the BGC of aurofusarin, the most well-known metabolite characterizing the red-colored *Cladobotryum* species (Milic *et al*. 2022), was located in scaffold 17 (Fig. 4). Aurofusarin is a dimeric polyketide belonging to the aromatic ones, also named naphthoquinones (Shibata *et al*. 1966). It was firstly found as a pigment in *Fusarium culmorum* (Ashley *et al*. 1937). The presence of aurofusarin's BGC was expected in *C. mycophilum* strain ATHUM6906, since a previous work in genus *Cladobotryum* showed that *C. mycophilum* is basal to the monophyletic clade formed by species producing this metabolite (Milic *et al*. 2022). The BGC of aurofusarin (Fig. 5) contained the polyketide synthase 1 gene (*pks1*) along with other genes encoding modification enzymes (*aur*O, *gip*1, *aur*F, *aur*J, and *aur*Z), aurofusarin biosynthesis cluster protein S (*aur*S), regulatory proteins (*aur*R1, *aur*R2), and a rubrofusarin-specific efflux pump (*aur*T; Hoffmeister and Keller 2007). According to relevant studies in the phytopathogenic fungus *F. graminearum*, aurofusarin is able to increase its competitive saprophytic ability because of its antibiotic properties, but it is not implicated in host colonization (Malz *et al*. 2005; Cambaza 2018).

Interestingly enough, the BGC cluster of a plant pathogenic metabolite, i.e. depudecin, was also retrieved in this mycophilic genome. Depudecin is an 11-carbon linear polyketide acting as an inhibitor of histone deacetylase (HDAC), which has been related with the virulence of the plant pathogenic fungus *Alternaria brassicicola* (Wight *et al*. 2009). The presence of this BGC prompts the question of whether it stands as an evolutionary vestige or if it plays a role in the mode of life of *C. mycophilum*.

### Comparative analysis of Hypocreaceae secondary metabolism

Comparative genomics were used to investigate the diversity and distribution of genes and BGCs responsible for the synthesis of secondary metabolites among 40 genomes of Hypocreaceae family (Table 1). High variation in gene cluster presence and absence was found among the genomes (Fig. 5). Among the 40 available genomes of species belonging to Hypocreaceae family, *C. mycophilum* exhibited the highest abundance of “core” secondary metabolic–related PCGs and in extent BGCs, followed by the rest of *Cladobotryum* species. Interestingly, in genomes of genus *Escovopsis*, which is phylogenetically positioned as sister clade to *Cladobotryum* spp., an extended contraction of BGCs was found, which is probably directly related with their small genome size (approximately 27 Mb), which is in accordance with the study of Scott *et al*. (2023), which showed that BGC abundance is correlated with genome size in *Trichoderma*.

Cluster network analysis (Big-SCAPE program) linked the BGCs of *C. mycophilum* with those of the other species under investigation, organizing these BGCs into families. Out of the 106 BGCs in *C. mycophilum*, 58 were found to be singletons. Overall, the BGCs found in the examined genomes were clustered in 3 categories according to their distribution, i.e. the common BGCs in all examined species, hereafter called category A, the “mixed” distribution (random existence) of BGCs, named here as category B, and finally, the BGCs that existed only in genus *Cladobotryum*, category C (Fig. 5).

T1PKS, NRPS and NRPS-like, terpene, and fungal RiPP BGCs were identified in all examined species, comprising category A (Fig. 5). In detail, T1PKS type included 537 BGCs (Supplementary Fig. 2). BGC network analysis organized them in 251 families including 172 singletons distributed in all examined species. The most abundant family was FAM_00378, which includes 16 BGCs of genus *Trichoderma*. Notably, some clusters exhibited a scattered distribution across various genera within Hypocreaceae, suggesting potential losses in several species. For instance, family FAM_01528 (Table 3), which comprises BGCs found in 2 *Trichoderma* species, 3 *Cladobotryum* species, and *H. perniciosus*, was similar to the linear tetracyclic aromatic polyketide naphthacenedione “TAN-1612” cluster, originally identified in *Aspergillus niger* (Li *et al*. 2011). Conversely, certain T1PKS families were exclusively identified within *Cladobotryum* species, like FAM_01527 and FAM_01550, which are implicated in the production of depudecin and cichorine, respectively (Wight *et al*. 2009; Ahuja *et al*. 2012; Sanchez *et al*. 2012). A total of 723 NRPS BGCs were identified in the studied species (Supplementary Fig. 2). The largest family, FAM_00369, encompassed 16 BGCs found in *Sphaerostilbella broomeana* and 15 *Trichoderma* species. NRPS FAM_01191 (Table 3) included *Cladobotryum* species and *H. perniciosus*, sharing a 50% identity with the chrysogine BGC in *F. graminearum* (Wollenberg *et al*. 2017). Families FAM_01459, FAM_01615, FAM_0124, and FAM_01271 (Table 3) exclusively comprised BGCs from *Cladobotryum* species and exhibited similarities to BGCs responsible for producing the cyclic tetrapeptide apicidin, pyranonigrin E, swainsonine, and ascochlorin, respectively (Jin *et al*. 2010; Andersen *et al*. 2011; Cook *et al*. 2017; Araki *et al*. 2019). A total of 299 BGCs were associated with the biosynthesis of terpenes, and they were classified into 132 families, with 93 of them being singletons (Supplementary Fig. 2). While FAM_00237 (Table 3) represented the largest terpene family, comprising 23 BGCs from all Hypocreaceae genera, the specific terpene metabolite production associated with it could not be identified. Finally, a total of 181 BGCs were identified as fungal RiPPs, categorized into 122 families, with 92 of them existing as singletons (Supplementary Fig. 2). FAM_01311, a fusion of a fungal RiPP and NRPS, emerged as the most populous family, comprising 10 members exclusive to the genus *Trichoderma*, all sharing a 100% identity with the choline BGC from *Aspergillus nidulans* (Hai *et al*. 2019). *Cladobotryum mycophilum* possessed 3 RiPP BGCs, with 1 belonging to FAM_01522 (Table 3), aligning with the respective BGC of *C. dendroides*, and 2 as standalone singletons. Nevertheless, it was not possible to determine the potential products generated by *C. mycophilum* RiPPs due to lack of reference data.

**Table 3.** Overview of BGCs in *C. mycophilum* ATHUM6906, detailing their type and genomic location.

Number	BGC	Type	Location [from–to]	Family	Most similar known cluster (MIBiG accession)
1	contig1.region001	T1PKS	Contig 1 [20,937–68,853]	FAM_01521	
2	contig1.region002	Fungal RiPP	Contig 1 [204,455–246,483]	FAM_01522	
3	contig1.region003	T1PKS	Contig 1 [1,036,058–1,079,067]	FAM_01523*^a^*	
4	contig1.region004	NRPS-like, terpene	Contig 1 [1,427,122–1,468,197]	FAM_01524*^a^*	EQ-4 (BGC0001668)
5	contig1.region005	NRPS	Contig 1 [1,470,220–1,514,188]	FAM_01525	
6	contig1.region006	NRPS-like	Contig 1 [1,721,962–1,761,887]	FAM_01526*^a^*	
7	contig1.region007	T1PKS	Contig 1 [4,897,237–4,943,226]	FAM_01527	Depudecin (BGC0000046)
8	contig1.region008	T1PKS	Contig 1 [4,994,689–5,030,604]	FAM_01528	
9	contig1.region009	Terpene	Contig 1 [5,138,026–5,155,781]	FAM_01529	
10	contig1.region010	T1PKS, terpene	Contig 1 [5,158,976–5,238,693]	FAM_01530*^a^*	
11	contig1.region011	Terpene, NRPS	Contig 1 [5,266,986–5,324,387]	FAM_01531*^a^*	
12	contig1.region012	Terpene	Contig 1 [5,362,297–5,384,604]	FAM_01532*^a^*	
13	contig1.region013	NRPS, T1PKS	Contig 1 [5,470,781–5,522,004]	FAM_01533*^a^*	
14	contig10.region001	NRPS, T1PKS	Contig 10 [219,692–291,584]	FAM_01534*^a^*	TAN-1612 (BGC0000156)
15	contig10.region002	T1PKS	Contig 10 [368,207–413,059]	FAM_01535*^a^*	Neurosporin A (BGC0001668)
16	contig10.region003	NRPS	Contig 10 [460,675–503,357]	FAM_01536*^a^*	
17	contig10.region004	NRPS	Contig 10 [558,013–605,851]	FAM_01537*^a^*	Ustiloxin B (BGC0000627)
18	contig10.region005	Terpene	Contig 10 [1,278,927–1,300,991]	FAM_01514	
19	contig10.region006	NRPS	Contig 10 [1,334,843–1,379,470]	FAM_01539*^a^*	
20	contig10.region007	T1PKS	Contig 10 [1,606,196–1,651,183]	FAM_01540*^a^*	
21	contig10.region008	T1PKS	Contig 10 [2,199,826–2,243,385]	FAM_01541*^a^*	
22	contig10.region009	T1PKS	Contig 10 [3,716,849–3,748,753]	FAM_01542*^a^*	
23	contig10.region010	T1PKS, NRPS	Contig 10 [3,936,152–3,988,125]	FAM_01543*^a^*	
24	contig13.region001	NRPS-like	Contig 13 [24,41–69,081]	FAM_01544	
25	contig13.region002	NRPS, T1PKS	Contig 13 [95,903–196,559]	FAM_01545*^a^*	Chaetoglobosins (BGC0001182)
26	contig16.region001	Terpene	Contig 16 [272,867–293,287]	FAM_01546	
27	contig16.region002	T1PKS	Contig 16 [380,593–425,348]	FAM_01547*^a^*	
28	contig16.region003	T3PKS	Contig 16 [1,649,997–1,690,854]	FAM_01548*^a^*	
29	contig16.region004	NRPS	Contig 16 [1,980,369–2,022,000]	FAM_01191	
30	contig16.region005	T1PKS	Contig 16 [2,179,010–2,222,991]	FAM_01550	Cichorine (BGC0000037)
31	contig16.region006	Terpene	Contig 16 [3,599,879–3,610,426]	FAM_01205	
32	contig16.region007	NRPS	Contig 16 [3,972,935–4,023,015]	FAM_01206	
33	contig16.region008	T1PKS, NRPS	Contig 16 [4,292,252–4,339,457]	FAM_01553	Cytochalasin E/K (BGC0000983)
34	contig16.region009	T1PKS	Contig 16 [4,350,647–4,396,720]	FAM_01554*^a^*	
35	contig16.region010	T1PKS	Contig 16 [4,610,190–4,659,198]	FAM_01491	
36	contig16.region011	T1PKS, NRPS-like, NRPS, fungal RiPP	Contig 16 [4,744,292–4,896,083]	FAM_01556*^a^*	Imizoquin-A/-B/-C/-D|TMC-2A/-2B (BGC0001621)
37	contig16.region012	T1PKS, terpene	Contig 16 [4,913,043–4,982,503]	FAM_01493	
38	contig3.region001	NRPS	Contig 3 [302,615–351,334]	FAM_01558*^a^*	
39	contig3.region002	NRPS-like	Contig 3 [795,142–838,465]	FAM_01559*^a^*	
40	contig3.region003	Siderophore	Contig 3 [990,497–1,004,040]	FAM_01560*^a^*	
41	contig7.region001	Terpene	Contig 7 [59,882–70,098]	FAM_01561*^a^*	
42	contig7.region002	Fungal RiPP, terpene, T1PKS	Contig 7 [144,815–214,625]	FAM_01562*^a^*	Ustiloxin B (BGC0000627)
43	contig7.region003	NRPS	Contig 7 [461,278–502,739]	FAM_01563*^a^*	
44	contig7.region004	T1PKS	Contig 7 [586,782–624,931]	FAM_01564	Fumagillin|β-trans-bergamotene|fumagillol (BGC0001067)
45	contig7.region005	Terpene	Contig 7 [743,071–762,455]	FAM_01565*^a^*	
46	contig7.region006	T1PKS	Contig 7 [854,062–899,633]	FAM_01566*^a^*	
47	contig7.region007	Terpene	Contig 7 [1,795,286–1,816,474]	FAM_00237	
48	contig7.region008	Terpene	Contig 7 [2,166,178–2,178,846]	FAM_01137	Squalestatin S1 (BGC0001839)
49	contig7.region009	T1PKS	Contig 7 [2,484,124–2,529,280]	FAM_01448	Wortmanamide -A/-B (BGC0001954)
50	contig7.region010	T1PKS	Contig 7 [5,466,710–5,537,203]	FAM_01570	
51	contig7.region011	Terpene	Contig 7 [5,843,774–5,859,003]	FAM_01571*^a^*	
52	contig7.region012	Terpene, T1PKS	Contig 7 [6,069,426–6,119,692]	FAM_01572	Clavaric acid (BGC0001248)
53	contig7.region013	NRPS	Contig 7 [6,222,719–6,285,023]	FAM_01573	
54	contig7.region014	NRPS	Contig 7 [6,418,277–6,472,527]	FAM_01199	
55	contig7.region015	T1PKS	Contig 7 [6,960,878–7,008,551]	FAM_01575*^a^*	
56	contig7.region016	T1PKS	Contig 7 [7,041,422–7,089,522]	FAM_01576	Solanapyrone D (BGC0000146)
57	contig7.region017	NRPS	Contig 7 [7,536,613–7,583,924]	FAM_01577*^a^*	Chrysogine (BGC0001545)
58	contig8.region001	T1PKS, NRPS-like	Congit 8 [38,977–117,909]	FAM_01578*^a^*	
59	contig9.region001	T1PKS	Contig 9 [130,274–168,905]	FAM_01579*^a^*	
60	contig9.region002	Siderophore	Contig 9 [220,046–231,546]	FAM_01580*^a^*	
61	contig9.region003	T1PKS	Contig 9 [709,705–748,491]	FAM_01581	Alternariol (BGC0000013)
62	contig9.region004	Terpene	Contig 9 [753,035–766,04]	FAM_01260	
63	contig9.region005	NRPS, NRPS-like	Contig 9 [781,396–847,887]	FAM_01583	
64	contig9.region006	T1PKS	Contig 9 [2,328,638–2,372,546]	FAM_01584	
65	contig9.region007	Terpene	Contig 9 [3,448,248–3,465,323]	FAM_01585*^a^*	
66	contig9.region008	NRPS-like	Contig 9 [3,510,825–3,548,097]	FAM_01586*^a^*	
67	contig9.region009	T1PKS	Contig 9 [4,771,912–4,812,897]	FAM_01262	
68	contig9.region010	T1PKS	Contig 9 [5,251,051–5,297,281]	FAM_01588*^a^*	
69	contig9.region011	Indole	Contig 9 [5,715,029–5,736,413]	FAM_01589*^a^*	
70	contig9.region012	NRPS-like	Contig 9 [5,819,784–5,862,074]	FAM_01502	
71	contig9.region013	T1PKS	Contig 9 [5,919,206–5,978,029]	FAM_01591	
72	contig9.region014	NRPS, T1PKS	Contig 9 [6,129,121–6,199,192]	FAM_01592*^a^*	Leporin B (BGC0001445)
73	contig9.region015	NRPS	Contig 9 [6,205,218–6,267,181]	FAM_01593*^a^*	Destruxin A (BGC0000337)
74	contig9.region016	Terpene	Contig 9 [6,317,976–6,338,626]	FAM_01594*^a^*	
75	contig9.region017	NRPS-like, T1PKS, NRPS	Contig 9 [6,449,891–6,543,804]	FAM_01271	Ascochlorin (BGC0001923)
76	contig9.region018	T1PKS	Contig 9 [6,595,828–6,658,103]	FAM_01596*^a^*	
77	contig9.region019	T1PKS	Contig 9 [6,672,756–6,752,769]	FAM_01597*^a^*	
78	contig9.region020	NRPS	Contig 9 [6,801,947–6,848,793]	FAM_01598*^a^*	
79	scaffold12.region001	NRPS	Scaffold 12 [739,653–774,62]	FAM_01016	Dimethylcoprogen (BGC0001249)
80	scaffold12.region002	T1PKS	Scaffold 12 [792,59–838,101]	FAM_01600*^a^*	
81	scaffold12.region003	NRPS	Scaffold 12 [1,147,823–1,226,560]	FAM_01601*^a^*	
82	scaffold12.region004	NRPS, T1PKS	Scaffold 12 [1,278,228–1,361,597]	FAM_01231	
83	scaffold12.region005	NRPS, T1PKS	Scaffold 12 [1,716,476–1,767,455]	FAM_01230	
84	scaffold12.region006	NAPAA, NRPS	Scaffold 12 [2,898,411–2,951,408]	FAM_01604*^a^*	
85	scaffold12.region007	Terpene	Scaffold 12 [3,214,332–3,235,701]	FAM_01605*^a^*	
86	scaffold12.region008	T1PKS	Scaffold 12 [3,363,240–3,402,845]	FAM_01606*^a^*	
87	scaffold12.region009	T1PKS, NRPS	Scaffold 12 [3,418,472–3,502,365]	FAM_01607	
88	scaffold12.region010	NRPS	Scaffold 12 [3,729,321–3,784,772]	FAM_01459	Apicidin (BGC0000304)
89	scaffold12.region011	T1PKS	Scaffold 12 [3,809,533–3,850,712]	FAM_01609	
90	scaffold12.region012	NRPS-like, T1PKS	Scaffold 12 [3,869,049–3,916,556]	FAM_01247	Swainsonine (BGC0001793)
91	scaffold17.region001	T1PKS	Scaffold 17 [50,606–93,739]	FAM_01611*^a^*	
92	scaffold17.region002	NRPS	Scaffold 17 [113,167–165,135]	FAM_01481	
93	scaffold17.region003	T1PKS	Scaffold 17 [181,389–227,613]	FAM_01613*^a^*	
94	scaffold17.region004	NRPS	Scaffold 17 [1,490,267–1,553,136]	FAM_01480	
95	scaffold17.region005	T1PKS, NRPS	Scaffold 17 [1,664,879–1,744,092]	FAM_01615	Pyranonigrin E (BGC0001124)
96	scaffold17.region006	NRPS, T1PKS	Scaffold 17 [3,373,385–3,426,940]	FAM_01616*^a^*	
97	scaffold17.region007	NRPS, terpene	Scaffold 17 [3,451,272–3,512,235]	FAM_01617	
98	scaffold17.region008	T1PKS, NRPS	Scaffold 17 [3,837,995–3,950,986]	FAM_01618*^a^*	Verticillin (BGC0001820)
99	scaffold17.region009	T1PKS, NRPS	Scaffold 17 [4,151,159–4,228,823]	FAM_01475	
100	scaffold17.region010	NRPS-like	Scaffold 17 [4,307,093–4,364,974]	FAM_01620*^a^*	
101	scaffold17.region011	Other	Scaffold 17 [4,416,112–4,457,005]	FAM_01621*^a^*	
102	scaffold17.region012	Terpene	Scaffold 17 [4,503,384–4,504,782]	FAM_01622*^a^*	
103	scaffold17.region013	T1PKS	Scaffold 17 [4,566,858–4,615,436]	FAM_01623	Aurofusarin (BGC0002709)
104	scaffold17.region014	T1PKS, NRPS-like	Scaffold 17 [4,653,857–4,729,974]	FAM_01474	Naphthopyrone (BGC0000107)
105	scaffold17.region015	NRPS	Scaffold 17 [4,750,437–4,815,420]	FAM_01474	
106	scaffold17.region016	Terpene	Scaffold 17 [4,820,426–4,842,031]	FAM_01626*^a^*	

Their family classification is provided. Additionally, the most closely related known cluster and its corresponding MIBiG accession are shown.

^*a*^Singletons.

Category B included the beta-lactone, indole, and NRP metallophore BGCs since all of them presented a scattered distribution among Hypocreaceae (Fig. 5). However, based on the distribution of these BGCs in the genomes of the species analyzed in this work, it was found that some of these BGCs could not be related to the mycophilic mode of life, while the rest could potentially be involved. In specific, beta-lactone BGC was absent in *C. mycophium* but found in *Escovopsis*, *Mycogone* spp., and *T. harzianum* clades that include fungicolous species, comprising 3 different families, respectively (Fig. 5). Similar BGCs have been identified in other fungi, like *Stereum* spp., *Boreostereum vibrans*, *Cephalosporium* sp., *Fusarium* sp., *Scopulariopsis* sp., *A. niger*, and *Penicillum polonicum* (Robinson *et al*. 2019; Wen *et al*. 2020; Wang *et al*. 2022), while it was firstly isolated from the bacterium *Stenotrophomonas maltophilia* (Mercuri *et al*. 2002). The diversity of beta-lactone BGCs, along with their presence in nonmycophilic species, suggests that they may not be directly linked to the fungicolous mode of life. On the contrary, BGCs of indole compound production exhibit discontinuous distribution and may be related to the fungicolous mode of life since *Escovopsis weberi* can produce various compounds, including shearinine terpene-indole alkaloids, which have an impact on ant behavior (Batey *et al*. 2020). Additionally, it synthesizes diketopiperazines to counteract defensive bacteria and produces other small molecules that inhibit the fungal cultivar. Indolic-derived compounds have also been associated with plant growth promotion by *Trichoderma* spp. (Contreras-Cornejo *et al*. 2016). According to BGC network analysis, *Cladobotryum* indole BGCs were singletons, without similarity to any known BGC (Table 3). Finally, NRP metallophore BGC was spread among Hypocreaceae, and although it was found in *Cladobotryum* clade, this BGC was lost in *C. mycophilum*.

Interestingly, BGCs of NI-siderophore (i.e. NRPS-independent, IucA/IucC-like siderophore), T3PKS, and NAPAA were found only in *Cladobotryum* genomes (category C), with T3PKS BGC to be lost in *C. dendroides* (Fig. 5). Although a NI-siderophore BGC was identified, the related secondary metabolite is yet unknown. NAPAA BGC found in *Cladobotryum* spp. was characterized, and it was found that it was responsible for the production of the NAPAA, ε-poly-l-lysine, with 100% identity with the respective BGC of the endophytic fungus *Epichloë festucae* (Clavisipitaceae; Purev *et al*. 2020). Purev *et al*. (2020) identified this BGC and isolated the metabolite, proving the inhibitory activity of ε-poly-l-lysine against spore germination of the fungal and oomycete plant pathogens *Drechslera erythrospila*, *Botrytis cinerea*, and *Phytophthora infestans* (Purev *et al*. 2020). However, whether a similar mechanism was expected from *Cladobotryum* genomes against their fungal hosts remains a subject of future investigation, and thus, the present study could only hypothesize about this potential interaction.

Results of this comparative analysis became highly interesting when all PCGs comprising the examined BGCs of Hypocreaceae were analyzed, including genes encoding the core synthases, tailor enzymes, and BGC-specific transcription factors along with protective and hypothetical genes, and they were included in the orthologous comparative analysis (Supplementary Table 7). A total of 2,875 orthogroups were identified, but only 3 of them included PCGs from all the examined species. In detail, the orthogroup OG0000000 includes an LCL-type condensation (C) domain of NRPS, OG0000001 family 58-like fungal cytochrome P450 genes (CYP58, also known as Tri4 and trichodiene oxygenase), and OG0000002 genes encoding ABC-type multidrug transport system, with ATPase and permease components. Two hundred and seven orthogroups were found only in *Cladobotryum* species. Out of them, 93 were characterized and mapped against the GO database, and according to their molecular function, they could be clustered into 2 main groups (Supplementary Table 8). The first category included PCGs with catalytic activity (GO:0003824) and, in detail, isomerase (i.e. aspartate racemase activity), l-pipecolate oxidase, dioxygenase, acid–amino acid ligase, hydrolase acting on glycosyl bonds, and 5′ methylthioadenosine deaminase activities. The second category included PCGs, the binding properties (GO:0005488) on Flavin Adenine dinucleotide (FAD), metal ion, 4 iron and 4 sulfur clusters, and phosphorpantetheine.

Interestingly, OG0001232 orthogroup was annotated as necrosis-inducing secreted protein similar to the respective one, i.e. NIS1, found in phytopathogen *Colletotrichum orbiculare* (Yoshino *et al*. 2012), and according to GO mapping, it was targeted to host cell cytoplasm (C:GO:0005576; C:GO:0030430). In *C. mycophilum*, the respective PCG was included in the NRPS/T1PKS hybrid BGC identified in contig 10 (location: 3,987,099–3,987,744; total: 468 bp excluding introns). The produced protein is expected to interact with the lost receptor-like kinases (RLKs and RLCKs) inhibiting their activity, finally inducing host cell death (Irieda *et al*. 2019; Yoshino *et al*. 2012). But the question if the existence of this PCG is related with host pathogenicity and effectiveness of *Cladobotryum*'s fungicolous mode of life is still to be addressed in the future.

### Diversity and evolution of BGCs in Hypocreaceae

The distribution patterns of BGCs in Hypocreaceae genomes, as illustrated in Fig. 5, and the cluster network analysis suggested the possibility of new cluster formation, cluster loss, and HGT events. These findings also revealed interesting insights into the evolutionary history of secondary metabolite production in these fungi.

The presence of a significant number of singletons suggested that these singletons might confer unique or specialized metabolic pathways on *C. mycophilum*, setting it apart from the other examined species. Additionally, this phenomenon was not restricted only in *C. mycophilum*’s BGCs but also expanded in the other examined Hypocreaceae species, and it signified an ongoing rapid evolution of these BGCs, leading to the formation of entirely novel and distinct clusters. The diverse arrays of biosynthetic genes and their genetic variability could contribute to fungicolous ecological interactions and adaptations.

BGCs of category B, i.e. associated with beta-lactone, indole, and NRP metallophore compounds, exhibited a scattered distribution in the phylogenetic tree, indicating potential multiply independent gene loss or acquisition events over time. Furthermore, beta-lactone compounds have been identified not only in Hypocreaceae but also in various other fungal genera, such as *Fusarium*, *Cephalosporium*, *Aspergillus*, *Scopulariopsis*, and *Penicillium* (Liu *et al*. 2018; Robinson *et al*. 2019; Wen *et al*. 2020; Tang *et al*. 2022; Wang *et al*. 2022). Similarly, indole-based alkaloids have been reported not only in Hypocreaceae but also in other Hypocreales species such as *Ophiocordyceps xuefengensis* and *Clonostachys rosea* (Jiang *et al*. 2021; Qin *et al*. 2021). This wide distribution underscored the ecological importance of these compounds and their potential roles in shaping the interactions of fungi with their environments and other organisms. Their presence in other fungi, along with their scattered distribution within the Hypocreaceae family, strongly suggested that these clusters might have been lost multiple times during the evolutionary history of Hypocreales. This scenario of multiple cluster losses in different lineages highlighted the dynamic nature of fungal secondary metabolism and the plasticity of these genetic pathways over evolutionary time. It also indicated that the production of certain secondary metabolites might not provide a selective advantage under specific conditions, leading to the loss of the corresponding BGCs in certain fungal lineages.

Initially, there was limited genomic evidence of HGT in fungi BGCs (Rosewich and Kistler 2000). However, the noncontinuous distribution of BGCs among fungi raised doubts about their strict vertical inheritance. It has been suggested that in BGCs, the genes grouped together with linked functions may increase fungal fitness, because of the added advantage they provide in adapting to novel hosts after HGT (Slot 2017). The acquisition of BGC category C, i.e. the NI-siderophore, T3PKS, and NAPAA, only in *Cladobotryum* spp. raised the question if these clusters resulted from HGT. To investigate the origin of category C BGCs, phylogenetic tree construction for the core biosynthetic genes and AI analysis including all PCGs belonging to BGCs of category C were conducted (Supplementary Table 9).

In detail, an in-depth analysis revealed the identification of 2 NI-siderophore BGCs in *C. mycophilum* and *C. protrusum*, while *C. dendroides* harbored only 1, resulting in the characterization of 5 NI-siderophore BGCs in total. The phylogenetic tree constructed from this “NI-siderophore-matrix” showed their division into 2 distinct subgroups (Supplementary File 3). NI-siderophore BGCs belonging to subgroup A were identified in *C. mycophilum* and *C. protrusum*, but not in *C. dendroides*, while the NI-siderophore BGC found in genus *Cladobotryum* is designated as subgroup B. Although subgroup A was similar concerning its content as BGC between these 2 species, in *C. protrusum*, it was found to be partially overlapped by T1PKS and fungal RiPP–like clusters, while in *C. mycophilum*, it was located in between 2 T1PKS BGCs (Supplementary File 3). A Blastp search of both subgroups showed identity only with PCGs from non-Hypocreaceae species, which are not close relatives or phylogenetically linked with the members of this genus. An AI analysis was conducted to investigate the origin of all PCGs belonging to the 2 NI-siderophore BGCs. In subgroup A of NI-siderophore BGCs, 3 out of 5 PCGs were found to have foreign or indeterminate origins, including the PCG encoding the siderophore synthase. Notably, species within the genus *Aspergillus* appeared to be potential donors of these genes. The subgroup B of *Cladobotryum* NI-siderophore BGC included some genes, which have also been found in *Trichoderma* species. However, it is worth mentioning that the siderophore synthase and hydroxylase/desaturase genes lacked homologs within other species of the Hypocreaceae family. Instead, they exhibited the highest similarity to genes from the genus *Hirsutella*, which belongs to the Hypocreales order but in a different family (Ophiocordycipitaceae). This observation leads to intriguing questions about the origin of these genes (Supplementary Table 9). Specifically, it raises the possibility of HGT events, wherein these genes may have been acquired from potential donor species like *Hirsutella*. Alternatively, it prompts consideration of whether the presence of these genes exclusively in the *Cladobotryum* genus might be a result of extensive gene losses within the Hypocreaceae family (Supplementary File 3).

Similarly, in the case of T3PKS, BGCs were exclusively identified in the genomes of *C. mycophilum* and *C. protrusum*, but not in *C. dendroides* (Supplementary File 4). This cluster exhibits a similar structure in both species, and interestingly, in the case of *C. protrusum*, the T3PKS cluster partially overlaps with a T1PKS cluster, while in the case *C. mycophilum*, it is located among T1PKS and NRPS BGCs (Supplementary File 4). Due to these structural differences and BGC fusion, cluster network analysis classified T3PKS BGCs as singletons. After performing a phylogenetic tree analysis based on the “T3PKS-matrix” (Supplementary File 4), it became evident that the identified T3PKS genes in *Cladobotryum* were not closely related to any PCGs within the Hypocreaceae family. Instead, they showed a closer phylogenetic relationship with T3PKS from other members of the Hypocreales order, particularly with the respective genes of genera *Ophiocordyceps* and *Sarocladium*. Another observation worth mentioning was the presence of homologs of many additional biosynthetic and regulatory genes associated with the T3PKS BGC in the genus *Trichoderma* (Supplementary Table 9). However, it is important to note that in *Trichoderma*, these genes were not clustered within a single BGC, and some of them were not part of any contiguous BGC due to their lack of contiguity with core biosynthetic genes. Based on these findings, we propose that the T3PKS BGC likely originated through a combination of native biosynthetic and regulatory genes from the Hypocreaceae family, along with the incorporation of the core T3PKS gene, possibly through HGT with a non-Hypocreaceae donor-like species belonging to *Ophiocordyceps* or *Sarocladium*. This result aligns with a previous study that showed a scattered distribution of T3PKS in fungi due to HGT events (Navarro-Muñoz and Collemare 2020).

NAPAA, as described earlier, is considered a subgroup of NRPS-like BGCs responsible for producing NAPAAs, such as ε-poly-l-lysine (Purev *et al*. 2020). The NAPAA BGC was identified in the genomes of *C. dendroides* and *C. mycophilum*, while they were absent in *C. protrusum* (Supplementary File 5). This observation suggests that this BGC might have been acquired later in the evolution of the *Cladobotryum* genus. In both species in which NAPAA BGC was present, it overlapped with an NRPS cluster. Notably, in *C. dendroides*, this region additionally contained a T1PKS cluster. Due to the structural distinctions arising from the fusion of the *C. dendroides* BGC with a T1PKS cluster, the NAPAA BGCs were categorized as singletons. Through sequence similarity and phylogenetic analysis (see M&M:“NAPAA-matrix”), it was observed that the NAPAA core gene was closely related to NRPS-like genes found in various Hypocrealean species (Supplementary File 5). However, no similarity was found with species belonging to the Hypocreaceae family, except for a PCG of *S. broomeana*, which was characterized as NRPS-like. Interestingly, the clade containing NAPAA genes from *Cladobotryum* and *S. broomeana* was not monophyletic. It also included NRPS-like PCGs from other fungal genera, such as *Claviceps*, *Aspergillus*, *Metarhizium*, *Beauveria*, *Hirsutella*, *Ophiocordyceps*, and, as expected, the ε-poly-l-lysine synthetase of *E. festucae* (Purev *et al*. 2020; NCBI Acc. No. BBU42014). These findings align with the AI analysis of NAPAA gene, which revealed a null AI value among both Hypocrealean and non-Hypocrealean species (Supplementary Table 9). The presence of NAPAA BGCs in some *Cladobotryum* species and their close relationships with genes from diverse fungal lineages raises possibilities regarding the evolution of NAPAA biosynthesis. This phenomenon suggests 2 plausible scenarios: the occurrence of potential HGT events involving the acquisition of NAPAA BGCs or an alternative scenario characterized by extensive gene losses within Hypocrealean genera that could result in the presence of NAPAA BGC only in some taxa. Further research into the functions and roles of NAPAA BGCs in *Cladobotryum* and related fungi can provide valuable insights into the ecological adaptations and metabolic diversity of these organisms.

Therefore, the presence of numerous singletons, both in *C. mycophilum* and other Hypocreaceae species, suggests that these unique gene clusters may encode specialized metabolic pathways, showcasing ongoing BGC evolution and the potential emergence of novel metabolites. Additionally, the scattered distribution of category B BGCs in the phylogenetic tree implies multiple independent gene loss or acquisition events over time, reflecting the dynamic nature of fungal secondary metabolism. Some *Cladobotryum* BGCs, mostly of category C, unveil potential HGT events that enriched the arsenal of the organism’s secondary metabolites. Recent research has enlightened the advantages of incorporating core PCGs of secondary metabolites through HGT events, as demonstrated in cases such as *Thinopyrum elongatum* with its Fhb7 gene (Wang *et al*. 2020) and *Malassezia sympodialis* with its flavohemoglobin-encoding genes (Ianiri *et al*. 2020). In the former case, HGT from the endophytic *Epichloë* granted the plant the ability to deepoxidize trichothecenes produced by phytopathogenic *Fusarium* species (Wang *et al*. 2020). In the latter example, genes were incorporated into the *M. sympodialis* genome from diverse donor bacteria that are part of the mammalian microbiome, through independent HGT events, and facilitated the role of *M. sympodialis* as a predominant inhabitant of animal skin (Ianiri *et al*. 2020). In *Cladobotryum* species explored in this study, similar HGT events, which appear to be of a stochastic phenomenon in nature, contributed to the evolution of the *Cladobotryum* genus and purifying selection led to the survival of the best fit species. Nevertheless, the BGCs of category C cannot be directly correlated to *Cladobotryum*'s fungicolous mode of life without further experimental work. In other words, these HGT-acquired BGCs may directly or indirectly influence the fungicolous mode of life, yet their primary role appears to provide survival advantages to *Cladobotryum* species under other varying environmental conditions.

### Interphylum HGT

**Fig. 6.**
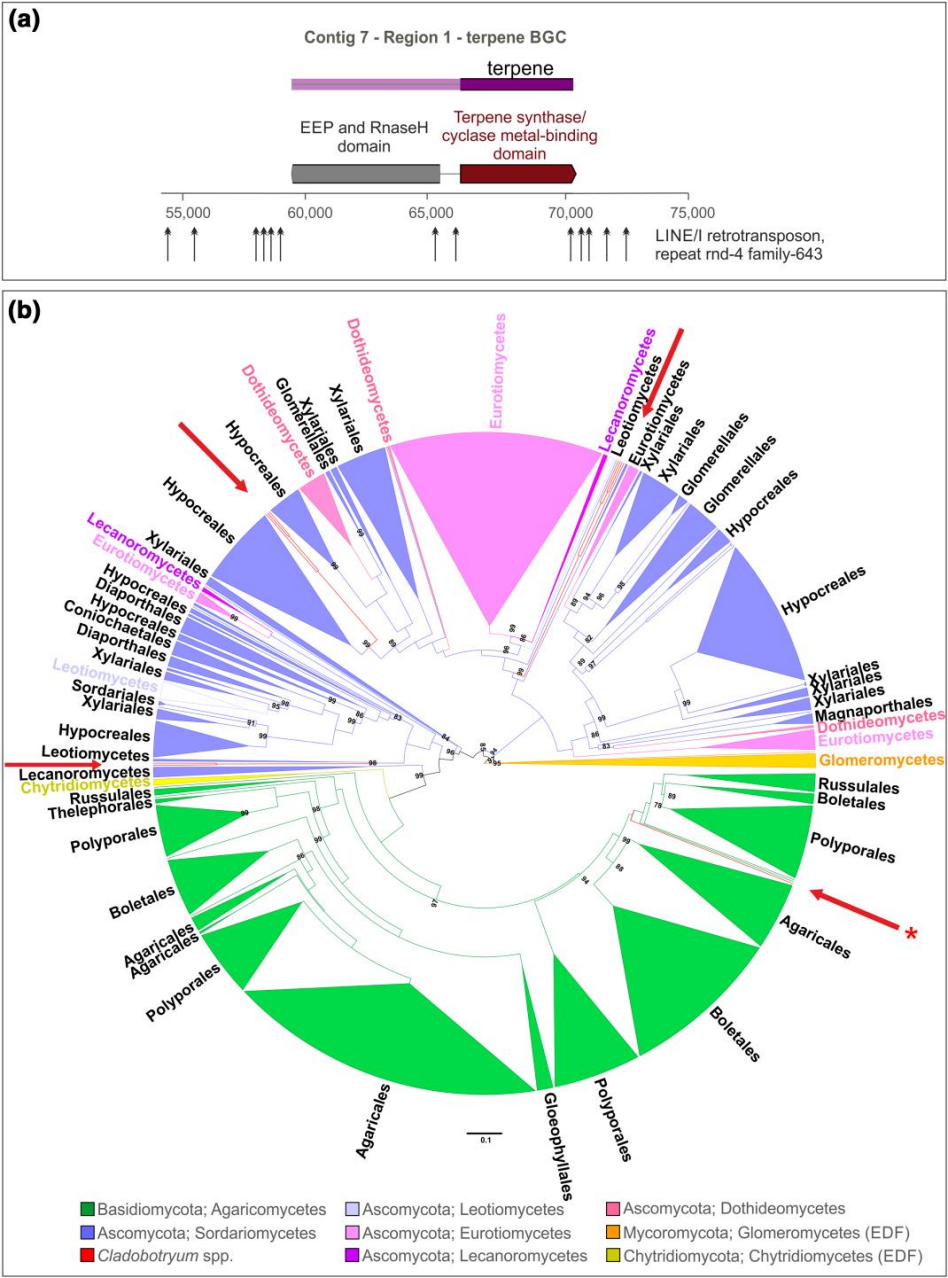
a) The BGC of the terpene BGC (region 1) located in contig 7. The BGC includes a synthase/cyclase with metal-binding domain core gene and upstream an EEP and RNaseH PCG. The locations of LINE/I retrotransposons (repeat rnd-4 family-643) within terpene BGC locus of contig 7 are also indicated with arrows. b) The terpene synthase phylogenetic tree produced by the ML method in IQ-TREE. Clades corresponding to Basidiomycetes, Sordariomycetes, Leotiomycetes, Eurotiomycetes, Lecanoromycetes, Dothideomycetes, Chytridiomycetes, and Glomeromycetes are also indicated. Clades indicated with arrows represent terpene synthases of *Cladobotryum* species. The core terpene synthase of region 1, contig 7 BGC (see detailed presentation in a), which is found among terpene synthases of Basidiomycetous origin, is indicated with an asterisk (*). The bootstrap values other than 100% are indicated in the nodes of the phylogenetic tree.

In addition to the earlier mentioned acquisition of BGCs in *Cladobotryum* species, a particularly interesting HGT event shaped the presence of a core PCG encoding a terpene synthase with a cyclase metal-binding domain. This terpene BGC (region 1), found within contig 7 of the *C. mycophilum* genome (Fig. 6a), captivated our attention due to its donor, which was identified as Basidiomycetes. Additionally, it was classified as a singleton by cluster network analysis. In-depth examination revealed that the phylogenetic analysis of all terpene synthase genes positioned this specific PCG within a clade exclusively comprising terpene synthases of Basidiomycetous origin (i.e. Agaricomycetes; Fig. 6b). In contrast, the remaining 3 *Cladobotryum* terpene synthases were positioned among Ascomycetes (Fig. 6b). According to the AI (AI = 88), this terpene synthase PCG seemed to be foreign and had emerged for HGT with Agaricomycetes donor (Supplementary Table 9). Notably, there was not any homologous gene present in the rest examined *Cladobotryum* genomes, indicating that this potential transfer was a recent event. Upstream and downstream of this gene, there was an rnd-4_family-643 repeat belonging to LINE/I retroelements (Fig. 6a). This observation indicated that the acquisition of this terpene synthase was facilitated by an HGT event involving a retrotransposon vector, during the later stages of *C. mycophilum*'s evolution. This event served as clear evidence of an ongoing and continuous interaction between mycophilic species and their hosts.

HGT is a mechanism believed to significantly shape genomes by rapidly introducing beneficial traits and expanding fungal metabolic capabilities, enhancing their adaptive strategies (Slot and Rokas 2010). Studies investigating the origin, evolution, and significance of gene clusters within fungal genomes, particularly focusing on the galactose utilization pathway, have demonstrated the independent emergence of these clusters in diverse lineages. These gene clusters play a pivotal role in fungal adaptation, enabling the dynamic acquisition and loss of metabolic capacities in response to changing environments (Slot and Rokas 2010). Furthermore, the acquisition of horizontally transferred genes in early-diverging fungal phyla, specifically those involved in nucleic acid synthesis and salvage, has been found to play a critical role in mediating interactions between the metabolic networks of hosts and pathogens (Alexander *et al*. 2016). Notably, other HGT events among Hypocrealean fungi and their hosts have been published. For instance, the evolutionary transition of *Metarhizium robertsii* from a plant endophyte ancestor to an insect pathogen has been linked to the acquisition of a sterol carrier gene through HGT from an insect (Zhao *et al*. 2014). Similarly, other examined HGT events, such as the transfer of high-affinity nitrate assimilation genes from Basidiomycetes to another Ascomycetous organism (*Trichoderma reesei*), underscore the vital role of HGT in facilitating the adaptation and success of Dikarya on land (Slot and Hibbett 2007). Collectively, these findings highlighted the significance of HGT in shaping fungal genomes, driving metabolic diversification, and contributing to their evolutionary fitness in changing ecological contexts. In the current case, the acquisition of a terpene synthase with Basidiomycetous origin did not seem to be related with any distinct advantage against *C. mycophilum* host, since the Basidiomycetous host carries a homologous PCG. Nonetheless, this event underscored the complex dynamics underlying the fungus–fungus interactions.

## Conclusion

The comprehensive examination of *C. mycophilum*'s whole genome, with an emphasis on the BGCs associated with secondary metabolism, has provided compelling insights into the distribution and potential loss of BGCs within fungal genomes of the Hypocreaceae family. Cluster network analysis indicated a dynamic evolution of BGCs in these fungi. The presence of a significant number of singletons in *C. mycophilum*’s BGCs suggested a level of genetic and metabolic diversity that might contribute to its ecological success and adaptability. Further, it was shown that *C. mycophilum* as well as the rest *Cladobotryum* species share unique BGCs, a phenomenon that can be elucidated by massive cluster losses or independent HGT events. In depth, the presence of the basidiomycetous origin terpene synthase underscored the complex dynamics of host–fungus interactions and the multifaceted role of HGT in shaping fungal genomes. These events appeared to deviate from a deterministic pattern governing the formation of BGCs that would unequivocally confer a survival advantage upon *Cladobotryum*. Instead, it is more likely that the assimilation of these acquired genes transpired in a stochastic manner, driven by neutral or positive selection pressures. However, further analyses including transcriptomics and metabolomics should be performed in the near future, to confirm if the horizontally acquired BGCs were related to *C. mycophilum*'s fitness in various environments and in extent in the fungicolous mode of life.

## Data Availability

This Whole Genome Shotgun project has been deposited at DDBJ/ENA/GenBank under the accession JAVFKD000000000. The provided BioProject and BioSample accessions for the genome of *Cladobotryum mycophilum*  ATHUM6906 are PRJNA933814 and SAMN33249331, respectively. Supplemental material available in Figshare (DOI: https://doi.org/10.6084/m9.figshare.23936796.v2).
